# Nuclear Morphometric Analysis (NMA): Screening of Senescence, Apoptosis and Nuclear Irregularities

**DOI:** 10.1371/journal.pone.0042522

**Published:** 2012-08-08

**Authors:** Eduardo C. Filippi-Chiela, Manuel M. Oliveira, Bruno Jurkovski, Sidia Maria Callegari-Jacques, Vinicius Duval da Silva, Guido Lenz

**Affiliations:** 1 Department of Biophysics, Federal University of Rio Grande do Sul (UFRGS), Porto Alegre, Rio Grande do Sul, Brazil; 2 Center of Biotechnology, Federal University of Rio Grande do Sul (UFRGS), Porto Alegre, Rio Grande do Sul, Brazil; 3 Institute of Informatics, Federal University of Rio Grande do Sul (UFRGS), Porto Alegre, Rio Grande do Sul, Brazil; 4 Graduate Program in Genetics and Molecular Biology, Department of Statistics, Federal University of Rio Grande do Sul (UFRGS), Porto Alegre, Rio Grande do Sul, Brazil; 5 Sao Lucas Hospital – Pontifícia Universidade Católica do Rio Grande do Sul (PUCRS), Porto Alegre, Rio Grande do Sul, Brazil; Enzo Life Sciences, Inc., United States of America

## Abstract

Several cellular mechanisms affect nuclear morphology which can therefore be used to assess certain processes. Here, we present an analytic tool to quantify the number of cells in a population that present characteristics of senescence, apoptosis or nuclear irregularities through nuclear morphometric analysis. The tool presented here is based on nuclear image analysis and evaluation of size and regularity of adhered cells in culture. From 46 measurements of nuclear morphometry, principal component analysis filtered four measurements that best separated regular from irregular nuclei. These measurements, namely aspect, area box, radius ratio and roundness were combined into a single nuclear irregularity index (NII). Normal nuclei are used to set the parameters for a given cell type, and different nuclear phenotypes are separated in an area versus NII plot. The tool was validated with β-gal staining for senescence and annexin or caspases inhibitor for apoptosis as well as several treatments that induce different cellular phenotypes. This method provides a direct and objective way of screening normal, senescent, apoptotic and nuclear irregularities which may occur during failed mitosis or mitotic catastrophe, which may be very useful in basic and clinical research.

## Introduction

The nucleus corresponds to approximately 10% of the cellular volume and, due to its nuclear envelope, presents a round shape and a well-defined and regular surface under normal conditions *in vitro*. Alteration in nuclear morphology occurs in physiologic situations, like during mitosis, and in several processes associated to cell death. These modifications include nuclear condensation and fragmentation observed in apoptosis, increase in nuclear size observed in senescence, and increase in nuclear irregularity observed in several conditions, such as chemical or physical stresses, defective activation or inactivation of cell cycle checkpoint signaling processes, or exogenous agents that affect microtubule dynamics or chromatin remodeling [1], [2].

Analysis of nuclear irregularities, such as mitotic catastrophe (MC), is limited almost exclusively to visualization of nuclei stained with DAPI (4′, 6-*diamino*-2-phenylindole), followed by subjective counting of nuclei with signs of MC. Recently, a tool was proposed for the detection of MC based on videomicroscopy of cells expressing markers of chromatin and centrosomes [3]. However, this tool requires specific equipment and is rather difficult to set up and analyze.

Both replicative senescence and oncogene-induced senescence are important anti-cancer mechanisms and reactivation of senescence by therapy may represent a good anticancer strategy [4], [5]. Senescence is analyzed by the level of β-galactosidase activity measured at pH 6.0 (known as senescent-associated β-galactosidase – SABG) [6], the formation of senescence-associated heterochromatin foci (SAHF) and the increase in the expression of proteins associated with senescence induction such as p21, p27, INK4A and Arf [7]. Senescence in culture cells produces a drastic increase in cellular and nuclear size, therefore a measure of nuclear size, associated with the reduction in mitotic cells, maybe a good indicator of the senescent state at a given cell culture population.

Induction of MC by small molecules or specific inhibition of DNA damage-activated signaling may increase cytotoxicity in cancer cells [8], [9]. In these cases, co-occurrence of senescence, MC and apoptosis is clinically desirable when compared to the occurrence of only one of these processes [10]. Therefore, a tool to easily evaluate morphological signs of apoptosis, MC and senescence is an important part in anti-cancer therapeutic studies.

Several cellular processes can be inferred by the analysis of nuclear morphometric features and a method without bias that evaluates nuclear phenotypes would widely improve our understanding concerning the dynamics of cell growth and death. Here, we developed an objective tool named Nuclear Morphometric Analysis (NMA) which is able to indicate the proportion of cells in senescence, apoptosis or with nuclear irregularities in a cell population *in vitro* based on nuclear morphology.

## Results

### 1. NMA Tool Development

In order to develop the tool we used nuclei from the glioblastoma cell line U87 treated with temozolomide (TMZ), which causes DNA damage and G_2_ cell cycle arrest, in combination with resveratrol, which blocks this arrest. This combination induced cells to undergo mitosis with increased expression of cyclin B1 and decreased pCDC2(Y15) levels, despite wide DNA damage, as indicated by γH2AX and comet assay (data not shown), thus generating a multitude of nuclear morphology. Nuclei were stained with DAPI and pictures were taken on a fluorescent inverted microscope. Images were analyzed with the Image Pro Plus 6.0 (IPP6) software and 46 parameters of nuclear size, shape and marking were produced for a large population of cells. These parameters were analyzed with principal component analysis (PCA) and the features Aspect (Asp), Area box (Arbx), Radius ratio (Rr) and Roundness (Rou) were combined in an index called Nuclear Irregularity Index (NII = Asp−Arbx+Rr+Rou). Details on the development of the tool are given in the **Text S1** file.

**Figure 1:**
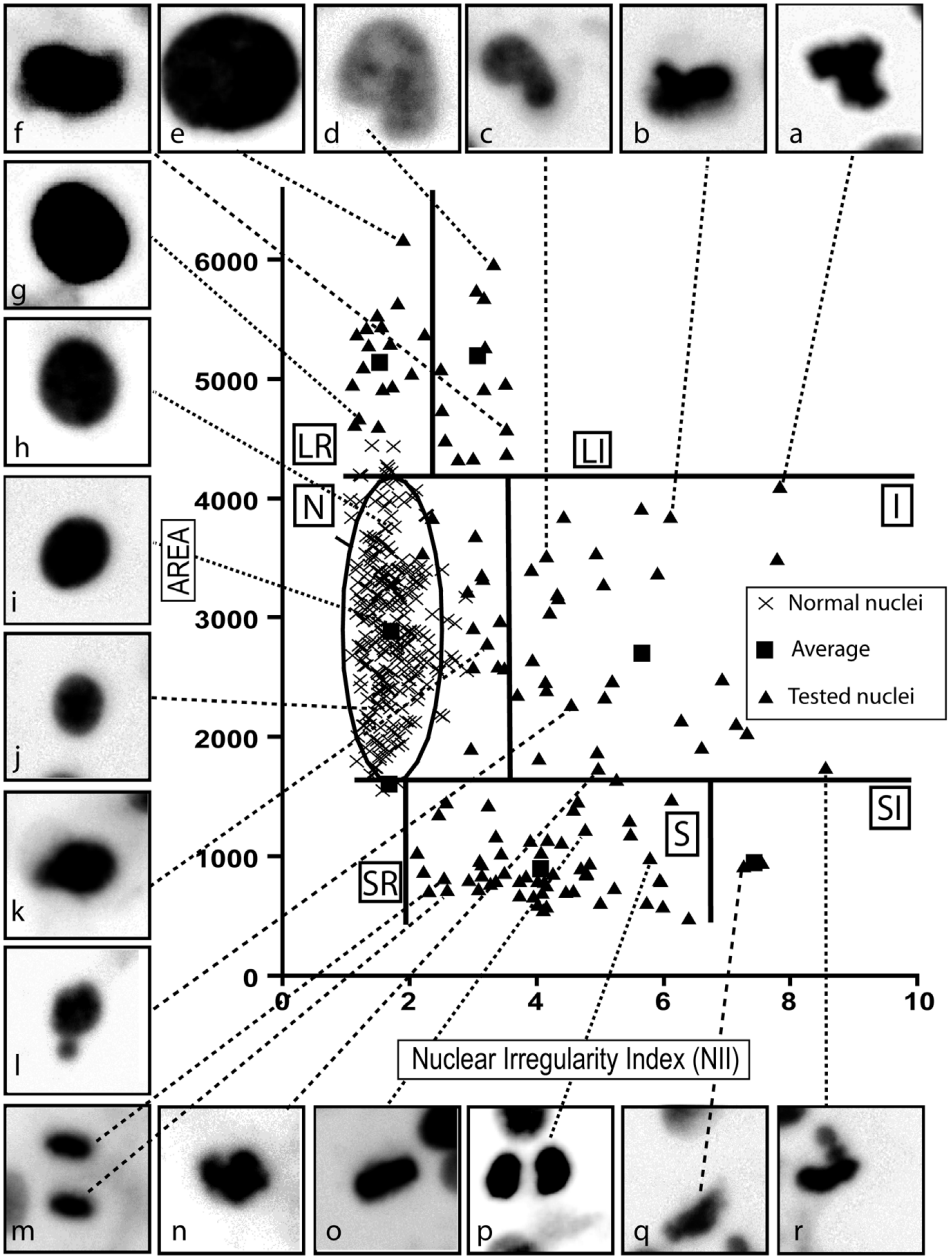
Distribution of nuclei in a plot of area versus NII. N: Normal nuclei (crosses represent nuclei used to establish the reference population and the ellipse that represents the conjoint distribution for area and NII for normal nuclei); I: irregular, LR: Large Regular; LI: Large Irregular; SR: Small and Regular; S: Small; SI: Small and Irregular. Squares denote the averages of the different populations. Pictures (a–r) show examples of nuclei and their localization in the graph.

In order to permit a wider use of the NMA tool, we developed an Image J plugin (for Image J 1.45 free version) that allows the quantification of the five measurements required for NMA (*i.e.* those for calculation of NII plus area measurement). A detailed explanation of the plugin features and its use is provided in the **Text S2** file. A comparative analysis of data generated with the IPP6 software and the Image J plugin that accompanies the NMA tool showed very similar percentages of nuclei for each population **(Text S1)**, validating the plugin developed for the NMA analysis.

**Table 1:** Morphological appearances and putative biological meanings of the different NMA populations presented in Figure 1.

Symbol	Name	Location in the graph	Morphological Appearance	Biological Meaning
N	Normal	Nuclei inside or close to the normal ellipse definedfrom a population of normal/regular nuclei	Normal shape and size	Interphase without damage thataffects nuclear morphology
I	Irregular	Nuclei with area similar to N nuclei, butwith high NII	Normal size and high irregularity	Mitotic catastrophe or othernuclear damaging event
SR	Small Regular	Nuclei with area below the normal ellipsewith low NII	Very condensed and regular	Apoptosis in an early orintermediary stage
S	Small	Nuclei with area lower than the normal ellipse,but with intermediate NII	Small, but not spherical	Mitosis
SI	Small Irregular	Nuclei with area lower than the normal ellipse,but with high NII	Condensed small and irregular	Mitosis with damage ornuclear fragments
LR	Large Regular	Nuclei with area above the normal ellipse,but with NII similar to N	Large and regular	Senescence
LI	Large Irregular	Nuclei with area above the ‘normal ellipse’,but with NII higher than that of N nuclei	Significant nuclear damage in largenuclei or large multi-nucleated cells	Mitotic catastrophe or othernuclear damaging event

Data generated with the IPP6 or Image J software were then analyzed in a spreadsheet (NMA.xls or NMA.ods). Data from normal, untreated cells (in which small, large and irregular nuclei are excluded) are used to set the parameters of the normal population. The user can define the number of standard deviations (SD) from the mean that will be considered an acceptable deviation. Next, a large set of data from different treatments are used to set the thresholds of the different populations. These settings (number of SD and the thresholds) are used to analyze the nuclei from different treatments. After entering the data from the nuclei in the data analysis sheets, a plot of area versus NII will be automatically produced (**Figure 1**), showing the ellipse representing the normal nuclei and the different populations whose thresholds were set in the “Normal Nuclei and Settings” sheet. Detailed use of the spreadsheets is given in the **Text S2** file. The Image J plugin, Microsoft Excel® and Open Office Calc files are available at http://www.ufrgs.br/labsinal/nma/.

**Figure 2:**
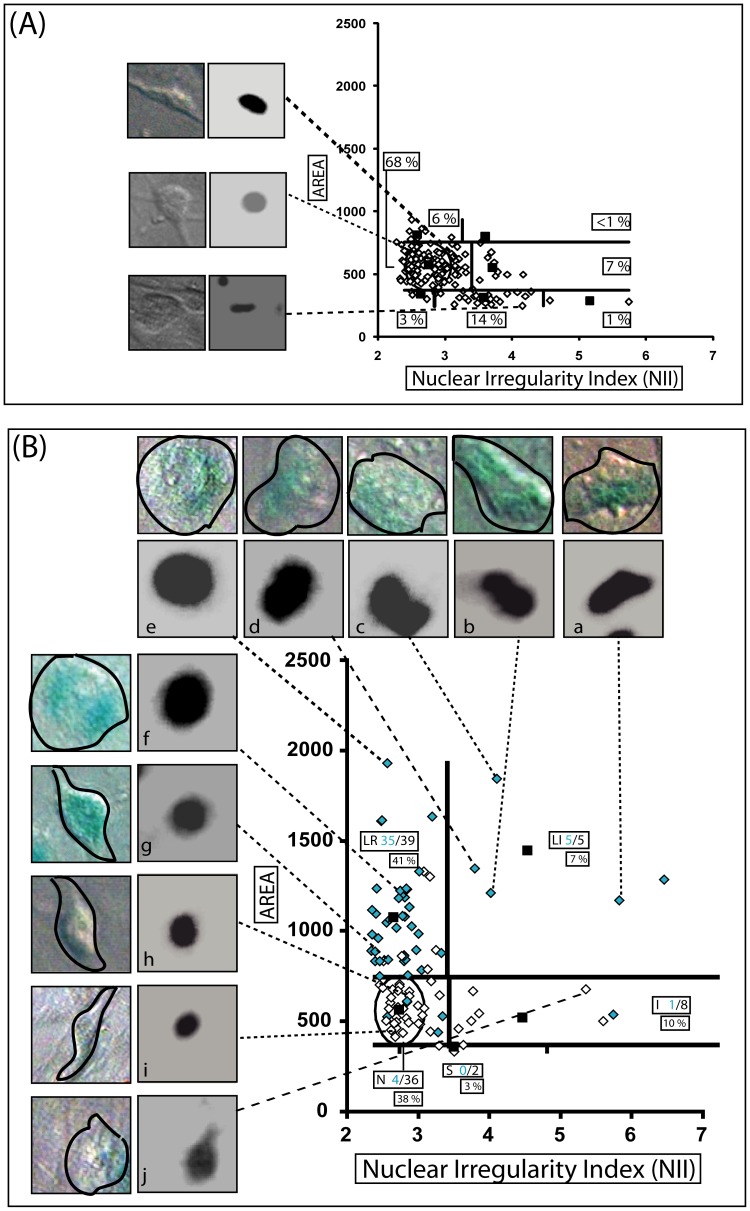
NMA of senescent glioma cells. C6 glioma cells were treated with DMSO as vehicle control (**A**) or resveratrol (Rsv) and quercetin (Quer) for 12 days (**B**) for senescence induction [6]. Light blue diamonds are nuclei from β-galactosidase positive cells. Numbers denote β-gal positive and total nuclei in the different categories and the percentage of nuclei in each category. Inserts show examples of nuclear and β-gal staining (a–g positive and h-j negative).

**Figure 3:**
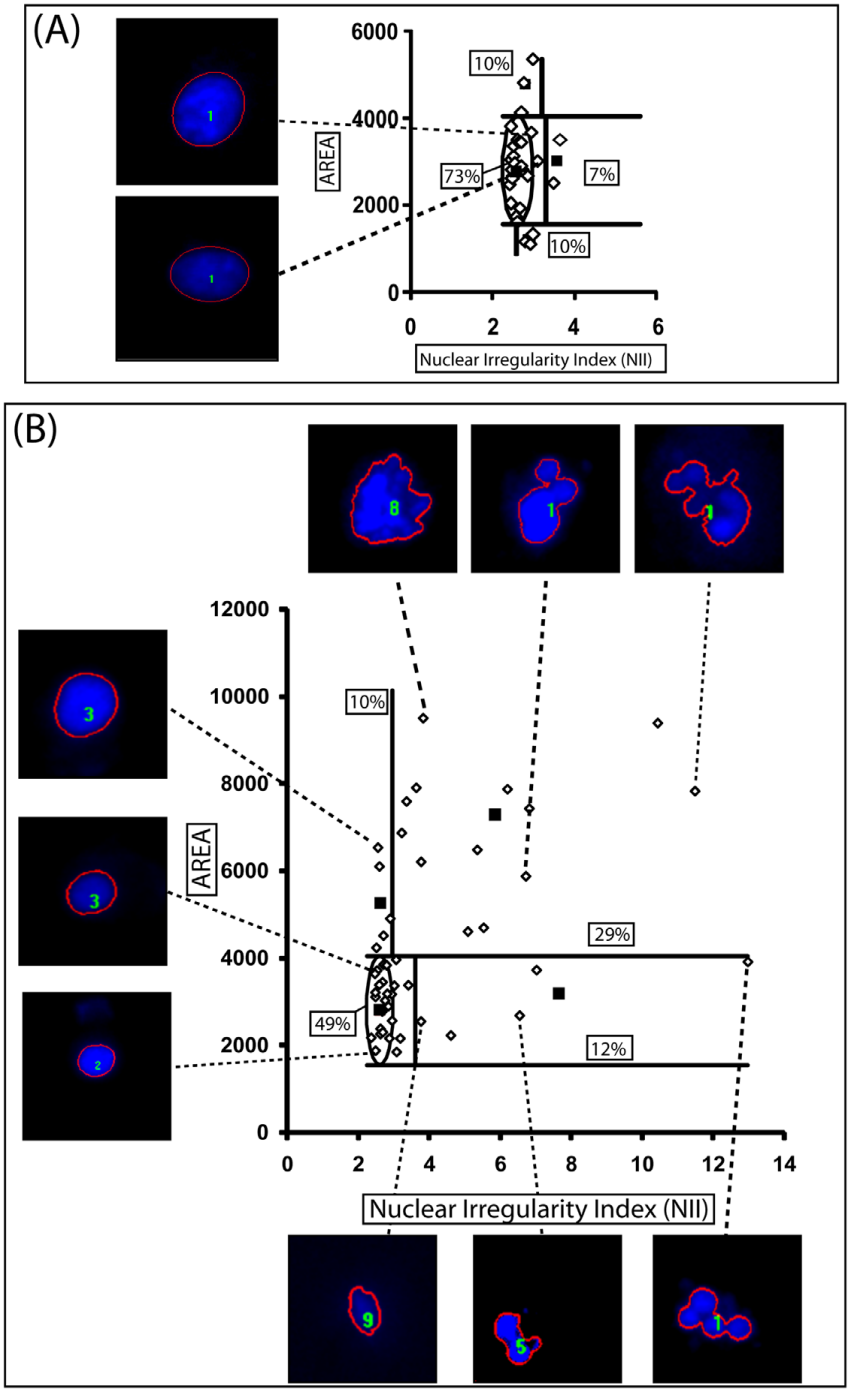
NMA ofcells treated with vincristine. U87 cells were treated with DMSO as vehicle control (**A**) or vincristine 50 nM for 24 h (**B**). Numbers in boxes denote the percentage of nuclei in each category. Inserts show examples of representative nuclei. Images were obtained directly from IPP6 software (with the boundary marking and the number of nuclei in the photo), to exemplify the marking established by the software. All nuclei are from single cells, as defined by direct visualization.

### 2. Biological Significance of the different Populations in the NMA Plot

Analyzing the data produced with the nuclear population used to set up the tool we noticed a distribution in four separate populations: normal, large, irregular and small (**Text S2**). Analysis of a larger set of conditions, including cells treated with classical inducers of senescence, apoptosis and nuclear irregularities, lead to the suggestion of seven different categories of nuclei based on size and NII (**Figure 1** **,** **Table 1** **and Text S1**).

Normal nuclei of adhered cells in culture vary in size, but have a very regular shape **(** **Figure 1** **.h,i,j)**, with some located at the borderline between N and I **(** **Figure 1** **.k)** and analysis of these nuclei are important for the precise setting of the threshold that separates these populations. Nuclei classified as I, **(** **Figure 1** **.a,b,c,l,n,r)** present high irregularity. Among the large nuclei, we found a clear separation between two groups in three experimental settings **(** **Figure 2** **,** **Figure 3** **and Figure S1 and S2)**. On the lower NII range are the well-defined and regular nuclear shapes **(** **Figure 1** **.e,g)**, whereas at higher NII range are the nuclei presenting morphological alterations **(** **Figure 1** **.d,f),** which may be undergoing MC.

**Figure 4:**
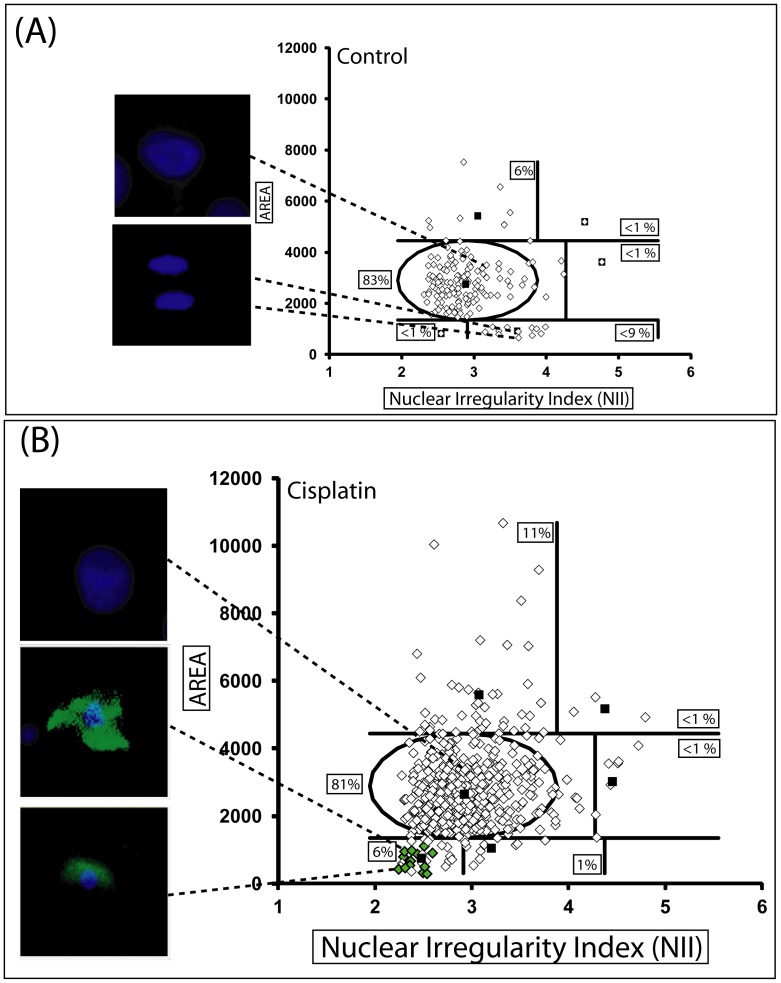
NMA of cells in apoptosis. U87 cells were treated with DMSO as vehicle control (**A**) (two representative overlaid images of nuclei and annexin are shown - both cells are negative for annexin) or Cisplatin (16.6 µM) (**B**) for 24 h, a well-established protocol of apoptosis induction [17] with three overlaid images of nuclei (blue staining) and annexin (green staining). Green diamonds on the graph represent annexin-positive cells.

**Figure 5:**
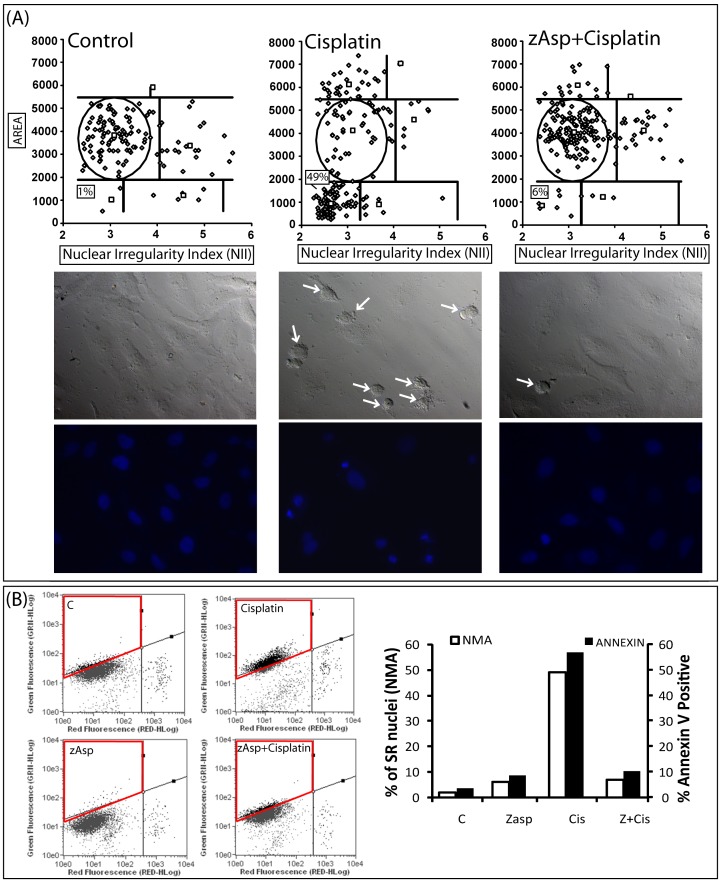
Comparison of NMA and annexin apoptosis quantification. (**A**) HeLa cells were pre-treated (1 h) with the caspases inhibitor zAsp-CH2-DCB (100 µM), followed by treatment with Cisplatin (40 µM), DAPI staining and NMA analysis. Top: NMA graphs; Middle: phase contrast; Bottom: DAPI fluorescence. Arrows point to cells with apoptotic phenotype. (**B**) HeLa cells were treated as in B, followed by annexin V-FITC staining and flow cytometry. Left: Flow cytometry plots. Green Fluorescence: annexin V; Red Fluorescence: PI. Region enclosed in red: Annexin-positive, PI-negative cells (*i.e.* apoptotic cells). Right: comparative percentage of apoptotic cells measured through NMA or annexin analysis.

Nuclei undergoing mitosis have higher values of NII than normal nuclei because of their elliptic shapes, and a broader range of NII due to the variations in the shapes of the mitotic plate **(** **Figure 1** **.m,p)**. However, some small nuclei also present very high irregularity **(** **Figure 1** **.q)**, being thus classified as small and irregular (SI). It is also important to stress that in the NMA tool, each mitotic cell will be counted as one before anaphase **(** **Figure 1** **.o)**, while after the anaphase the NMA tool will count two nuclei, which are normally located close to each other in the graph **(** **Figure 1** **.m)**. Therefore, the NMA is not a good tool for counting mitotic cells, since at anaphase the two nuclei counted will still be part of a single cell, and will be evaluated as two nuclei. Notwithstanding, it provides a broad assessment of mitotic proportion in a cell population, as illustrated by the almost complete absence of mitotic nuclei in the senescent population analyzed in **Figure 2**.

### 3. Senescent Nuclear Enlargement in the NMA Plot

For the validation of senescence, we used the rat glioma cell line C6 treated with the combination of resveratrol and quercetin, which we have previously shown to induce high levels of senescence [5]. About 90% (35 of 39) of the large nuclei(LR+LI) were β-galactosidase positive (blue diamonds and examples of positive cells in inserts **a–g**), whereas only 4 out of 36 nuclei that localized in the normal population were β-gal positive **(** **Figure 2** **– bottom graph)**. Additionally, in this population, only 2 mitosis were found in around 90 cells (around 2.2%), whereas in the control population this ratio was around 14% **(** **Figure 2** **– top graph)**. These data support the usefulness of the NMA tool in predicting the senescent status of a cell population, with the advantage of being much less subjective than the current standard method of β-gal staining and visual identification of the positive cells.

### 4. Nuclear Irregularities in the NMA Plot

To test the usefulness of the NMA tool to detect nuclear irregularities, we measured nuclei of a population of U87 glioma cells treated with vincristine (50 nM), which is described to induce several nuclear phenotypes, including MC and/or senescence [11], [12], depending on the cell type and treatment. We observed that the treatment induced an increase of irregular nuclei (I and LI), from 7% in control to 41% (12%+29%) **(** **Figure 3** **)**. Moreover, no nuclei were observed in the region were normal mitotic nuclei localized in the NMA graph.

In order to test the applicability of the NMA tool in other cell types, we treated the colorectal carcinoma cell line HCT116 with vincristine 50 nM. As expected, vincristine increased the percentage of both LI and LR nuclei. Average values of area of the LI and LR populations also increased, as did the NII of the LI population of nuclei treated with vincristine when compared to control **(Figure S1)**. It is important to note that induction of senescence-like phenotype in cancer cells usually is accompanied by MC [13]. Thus, our tool allows a dynamic analysis of the development of both events over time.

We have also performed an analysis of data from published papers of *Shao et al* [14] and *Vakifahmetoglu et al* [1], which presented enough control nuclei to set the standards for the studied cell line as well as pictures of nuclei undergoing MC that could be measured. Despite the small number of images for control nuclei, NMA provided a well-defined distribution for the control nuclei and a broad distribution of the catastrophic nuclei, mainly in the LI group. All treated nuclei localized in the I or LI regions of the NMA plot. This is expected, since the illustrative pictures probably were selected to clearly show the catastrophic nature of the nuclei **(Figure S2).**

### 5. Apoptotic Nuclear Condensation in the NMA Plot

Cells undergoing apoptosis suffer a high and regular condensation of the nucleus [15] which in culture occurs prior to nuclear fragmentation and cell detachment but almost concomitantly with the externalization of phophatidylserine and formation of blebbings in the cell membrane. Due to this high condensation in a near spherical form, we hypothesized that nuclei of cells undergoing apoptosis may appear as small and regular (SR), *i. e.* on the lower left corner of the NMA plot. In order to validate this hypothesis, we evaluated the induction of apoptosis in U87 cells treated with cisplatin the presence of zAsp, a caspases 3/7 inhibitor. Apoptosis was confirmed by annexin V-FITC staining. Fourteen out of 29 nuclei located in the SR were annexin V positive as visualized with a fluorescence microscope and none of the annexin V-positive cell was located in any region outside SR **(** **Figure 4B** **)**, whereas no cell was annexin V-positive in the vehicle-treated **(** **Figure 4A** **)**.

A side by side analysis with NMA and annexin analysis through flow cytometry presented a very good correlation **(** **Figure 5B** **)**. Inhibition of apoptosis with a caspases inhibitor reduced the apoptotic population detected with NMA to 7% and annexin to 10% in comparison to 49 and 57%, respectively, in the absence of zAsp **(** **Figure 5A and B** **)**.

Additionally, NMA was also able to detect etoposide-induced apoptosis in HeLa cells, as well as differentiate apoptotic and mitotic cells **(Figure S3)**, which normally appear in the S region of the NMA graph. Precise separation of S and SR regions may difficult the quantitative analysis of mitotic cells using NMA and additional methods should be employed to confirm mitotic cells. We also performed a kinetic analysis of Hoechst 3342-stained nuclei of HeLa cells treated with cisplatin. NMA graphs with the dynamics of area and NII, together with visible images of the cells undergoing apoptosis support the notion that nuclei located in the SR region of the NMA graph are apoptotic independent of the size and shape of the nuclei before apoptosis **(Figure S4)**.

## Discussion

We present a new and objective tool based on measurements of morphometric data that allows the unbiased simultaneous analysis of nuclear features indicative of normal, senescent, apoptotic and irregular nuclei. It is important to stress that a method based solely on nuclear morphology is simplistic for the definitive identification of such complex cellular processes. Final identification of these processes has to be confirmed with standard techniques for each individual process. However, this is in line with the current way of detecting complex cellular states, which require more than one marker for definitive identification. Notwithstanding, the ability to easily identify these processes simultaneously gives this tool the advantage of an overview of the population dynamics related to these processes.

There is a need for the development of objective, computer generated, analysis of morphological information of cells and tissues. Recently, a tool to predict the outcome of breast cancer patients based on morphometric analysis of tissues performed by a software developed by a machine learning-based method far outmatched other methods for predicting survival [16], indicating the potential for computer-based morphometric analysis.

It is also important to point out that the NMA tool is an easy to use and objective quantification of senescence and nuclear irregularities of adhered cells in culture, two processes normally assessed by subjective analysis of morphology or intensity of β-galactosidase labeling, respectively. Therefore, we believe that this tool can provide important new insights in the biology of complex cellular populations under normal and, most importantly, stressful conditions both in basic cell biology and, potentially, in pathological analysis.

## Methods

### 1. Software Information

Protocol uses Image Pro Plus 6.0 (IPP6 - Media Cybernetics, Bethesda, MD, USA), Image J 14.45 (NHI, Bethesda, MD, USA; http://rsbweb.nih.gov/ij/), PASW Statistics 18.0 (formerly known as SPSS - http://www.spss.com/software/statistics/), Microsoft Excel 2003 or 2007 (Microsoft, Redmond, WA, USA) and Open Office Calc (www.openoffice.org). NMA plugin, spreadsheets and sample images are available at http://www.ufrgs.br/labsinal/nma/.

### 2. Procedure

Brief instructions for using the NMA procedure (see **Text S2** file for detailed instructions) are:

1. Take DAPI images (with at least 300 dpi; preferentially.TIFF format) of at least 100 nuclei in each condition of experiment; do not take images of fields in high confluence;
2. Open an image on IPP6 or Image J (containing the plugin of NMA) and correctly mark the surrounding of nuclei to assess morphometric data to NMA, *i.e.* area, aspect, area box, radius ratio and roundness;
3. After obtaining raw data, choose a group of nuclei from the control condition and exclude nuclei in mitosis or with clear abnormalities. Use the data from these nuclei to set the parameters for the normal population. Paste data from these nuclei in the spreadsheet named *“Normal Nuclei and Settings”*, from NMA file from Excel or Open Office; choose the number of SD that best defines the ‘normal ellipse’; if using Excel, don’t forget to erase the formula in blank cells of the NII column;
4. Paste data from all conditions - control and for each treatment - into the columns named “Treated Nuclei (for setting)”, in the *“Normal Nuclei and Settings”* spreadsheet of NMA file from Excel or Open Office; thereafter, choose the number of SD to define the thresholds that better separate the populations;
5. Create as many spreadsheets as needed (one to each condition) and paste data from analyzed nuclei into them;
6. Analyze the percentage of nuclei in each population as well as the averages of the populations.

### 3. Cell Lines

U87, C6, HeLa and HCT116 cell lines were purchased from ATCC (American Type Cell Collection, ATCC, Rockville, MD) and were maintained in DMEM supplemented with 10% FCS in 5% of CO_2_ at 37°C.

### 4. Annexin Staining

Briefly, cells were pre-incubated with zAsp or vehicle for 1 h, followed by cisplatin treatment for 24 h. After this, for flow cytometry analysis, supernatant and cells were harvested, washed once with PBS 1x and once with annexin binding buffer, and incubated with a solution containing 6 µM of PI and 0.5 µL *per* sample of annexin V-FITC (Sigma Chemical; St. Louis, MO, USA) for 30 min, as indicated by the manufacturer, followed by flow cytometry. For microscopy, cells were washed once with PBS 1x and once with annexin binding buffer, and incubated with a solution containing 6 µM of PI and 1 µL of annexin *per* sample for 30 min, as indicated by the manufacturer. After, cells were fixed with paraformaldehyde 2% in PBS, stained with DAPI and images were acquired and overlaid.

## Supporting Information

Figure S1 **NMA of colon cancer cells treated with vincristine.** HCT116 colon cancer cells were treated with DMSO as a vehicle control **(A)** or vincristine 50 nM **(B)** for 24 h. Numbers in boxes denote the percentage of nuclei in each category as indicated and averaged area and NII for LR and LI nuclei.
(TIF)

Figure S2 **NMA of published MC nuclei.** Images of normal and MC cells from published papers were analyzed using NMA [1], [14].
(TIF)

Figure S3 **NMA of HeLa cells treated with cisplatin or etoposide.** HeLa cells were treated with DMSO as vehicle control, cisplatin (40 µM) or etoposide (100 µM) [17], for 24 h, followed by fixation and image acquisition. **(A)** Overlaid images of cells (visible) and DAPI-stained nuclei. SR – small and regular nucleus; S – small nucleus; N – normal nucleus. **(B)** NMA plots of the treatments. Red diamonds represent cells with a mitotic morphology.
(TIF)

Figure S4 **Dynamic nuclear condensation measured by NMA.** HeLa cells were treated with cisplatin (40 µM) for 18 h. At this time, the same fields were photographed every hour, during 5 hours. **(A)** NMA of three consecutive measurements one hour apart. Left: normal nuclei; right: apoptotic nuclei. Numbers correspond to the nuclei in (B). **(B)** Phase contrast and fluorescent images of live cells stained with Hoechst 33342. Arrows point to nuclei that suffer a strong nuclear condensation.
(TIF)

Text S1 **Supporting Results.** This file contains details of the development of the NMA tool and a detailed comparison between Image J and IPP software.
(DOC)

Text S2 **Supporting Methods.** This file describes the instructions of using Image J, IPP and Spreadsheets to perform the NMA analysis.
(DOC)
