# Supplementary figures and images for: Nuclear Morphometric Analysis (NMA): Screening of Senescence, Apoptosis and Nuclear Irregularities
Source: PLoS One. 2012 Aug 8;7(8):e42522. doi: 10.1371/journal.pone.0042522 (PMC3414464; doi:10.1371/journal.pone.0042522)

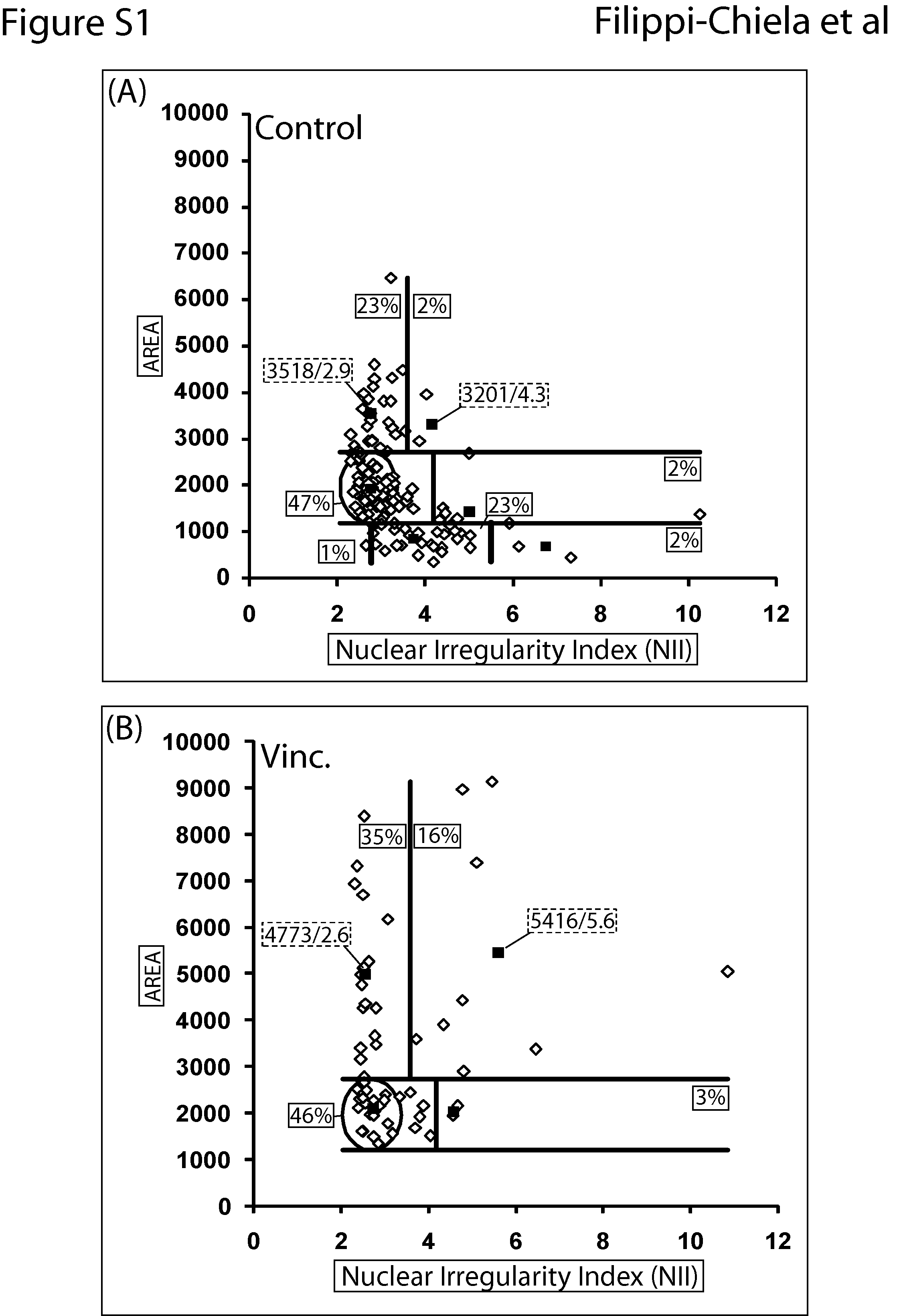

Supplement: Figure S1 — NMA of colon cancer cells treated with vincristine. HCT116 colon cancer cells were treated with DMSO as a vehicle control (A) or vincristine 50 nM (B) for 24 h. Numbers in boxes denote the percentage of nuclei in each category as indicated and averaged area and NII for LR and LI nuclei. (TIF) [file pone.0042522.s001.tif]

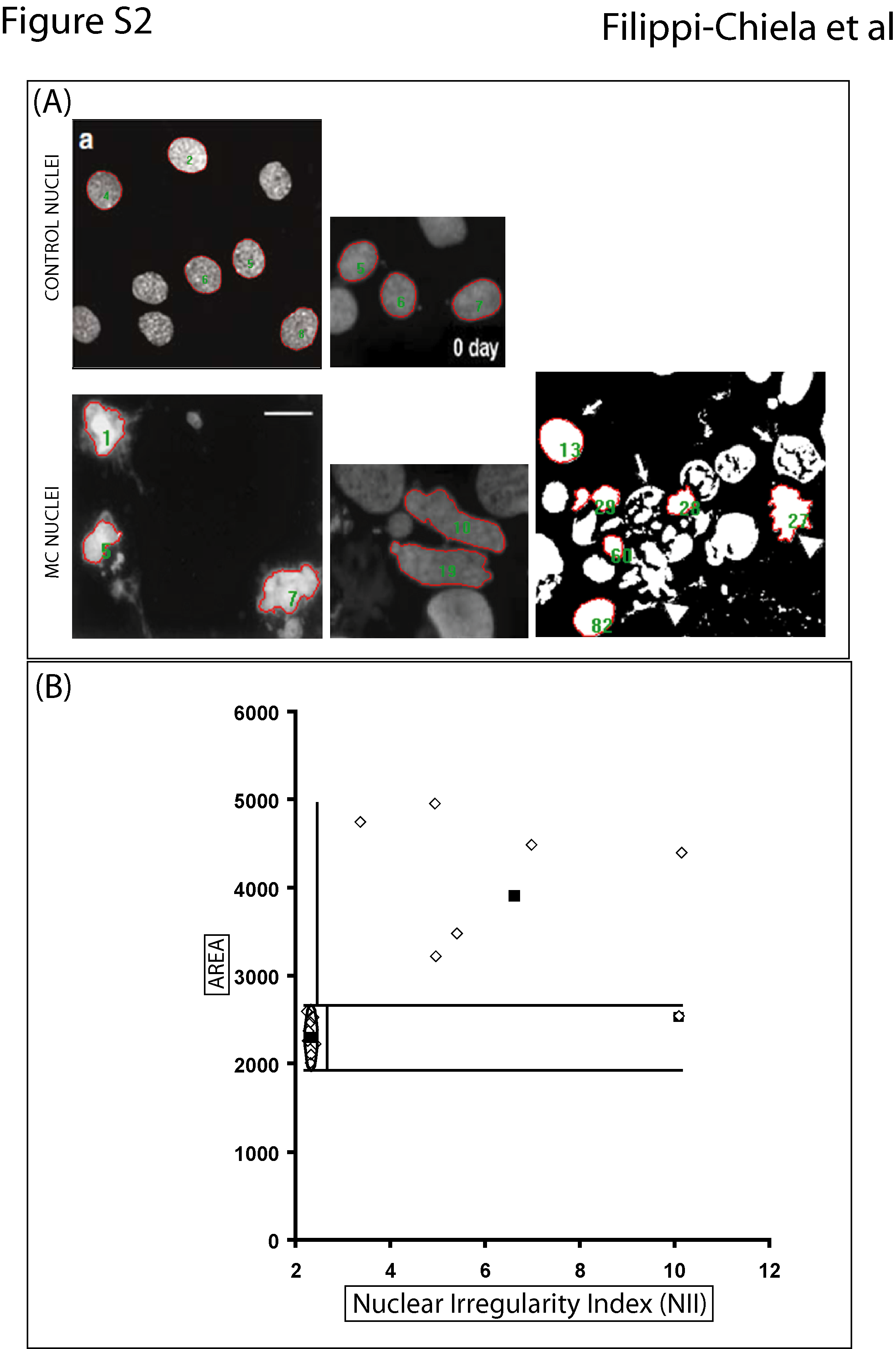

Supplement: Figure S2 — NMA of published MC nuclei. Images of normal and MC cells from published papers were analyzed using NMA [1], [14]. (TIF) [file pone.0042522.s002.tif]

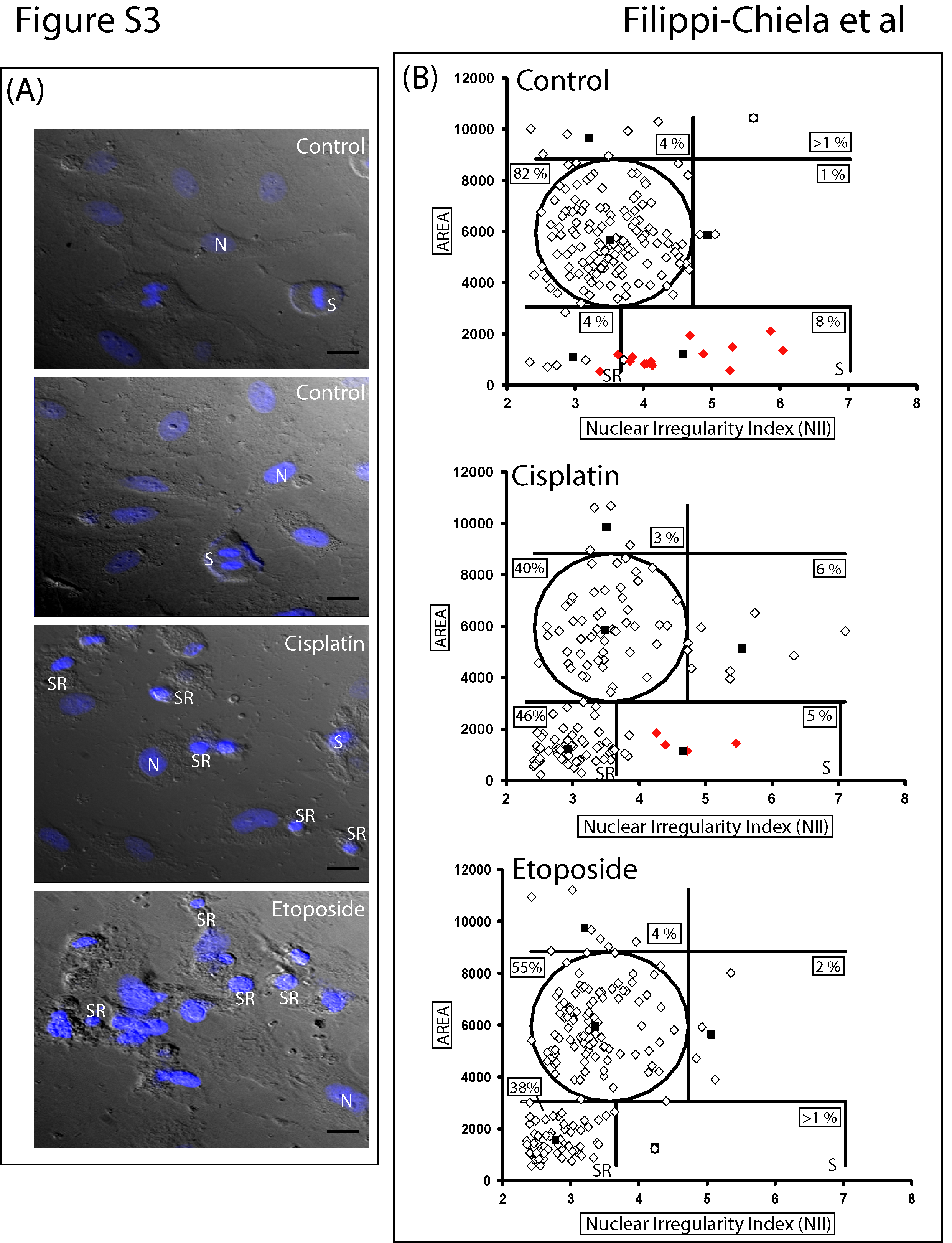

Supplement: Figure S3 — NMA of HeLa cells treated with cisplatin or etoposide. HeLa cells were treated with DMSO as vehicle control, cisplatin (40 µM) or etoposide (100 µM) [17], for 24 h, followed by fixation and image acquisition. (A) Overlaid images of cells (visible) and DAPI-stained nuclei. SR – small and regular nucleus; S – small nucleus; N – normal nucleus. (B) NMA plots of the treatments. Red diamonds represent cells with a mitotic morphology. (TIF) [file pone.0042522.s003.tif]

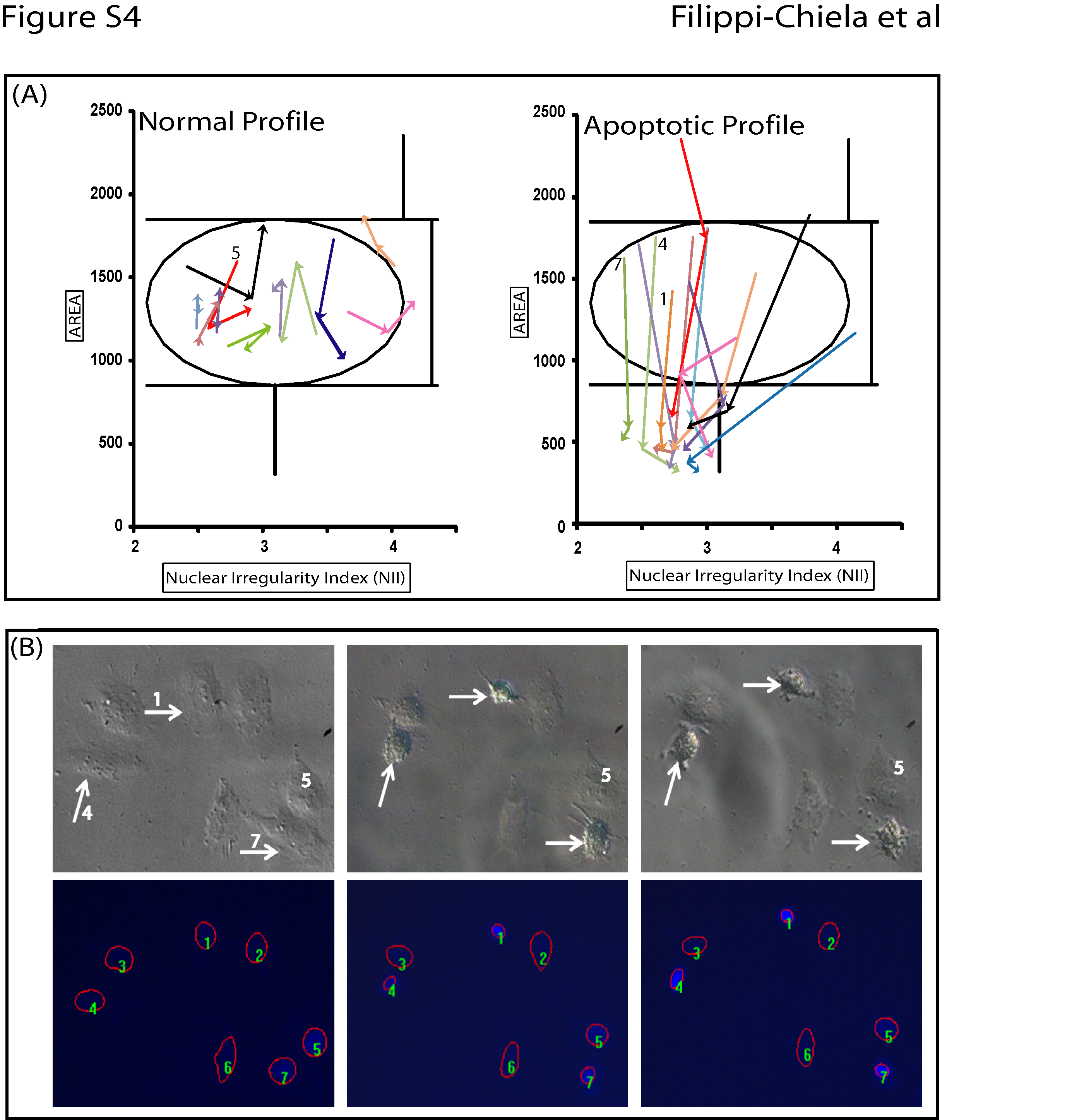

Supplement: Figure S4 — Dynamic nuclear condensation measured by NMA. HeLa cells were treated with cisplatin (40 µM) for 18 h. At this time, the same fields were photographed every hour, during 5 hours. (A) NMA of three consecutive measurements one hour apart. Left: normal nuclei; right: apoptotic nuclei. Numbers correspond to the nuclei in (B). (B) Phase contrast and fluorescent images of live cells stained with Hoechst 33342. Arrows point to nuclei that suffer a strong nuclear condensation. (TIF) [file pone.0042522.s004.tif]
