# Supplementary material for: Nuclear Morphometric Analysis (NMA): Screening of Senescence, Apoptosis and Nuclear Irregularities
Source: PLoS One. 2012 Aug 8;7(8):e42522. doi: 10.1371/journal.pone.0042522 (PMC3414464; doi:10.1371/journal.pone.0042522)
Supplement: Text S1 — Supporting Results. This file contains details of the development of the NMA tool and a detailed comparison between Image J and IPP software. (DOC) [file pone.0042522.s005.doc]

**Nuclear Morphometric Analysis (NMA):**

**Screening of apoptosis, mitosis, senescence and mitotic catastrophe**

Eduardo C. Filippi-Chiela^1^, Manuel M. Oliveira^3^, Bruno Jurkovski^3^, Sidia Maria Callegari-Jacques^4^, Vinicius Duval da Silva^5^; Guido Lenz^1,2^,*

# Supporting Results

**_________________________**

**Principal Component Analysis (PCA) for NMA**

To develop the tool we used a population of nuclei from the glioblastoma cell line U87 treated with Temozolomide (TMZ), which causes DNA damage and G_2_ cell cycle arrest, combined with a drug that blocks this arrest and makes the cells undergo mitosis with wide DNA damage, thus generating a multitude of nuclear morphology that is characteristic of mitotic catastrophe (MC).

Nuclei were stained with DAPI and pictures were taken on a fluorescence microscope. Images were analyzed using the IPP6 software (as described in *Supporting Methods file*) and 46 parameters of nuclear size, shape and marking **(Supp. Results Table 1)** were produced for a large number of nuclei. This data was used for the statistical analysis of measurements of main significance for morphometric analysis.


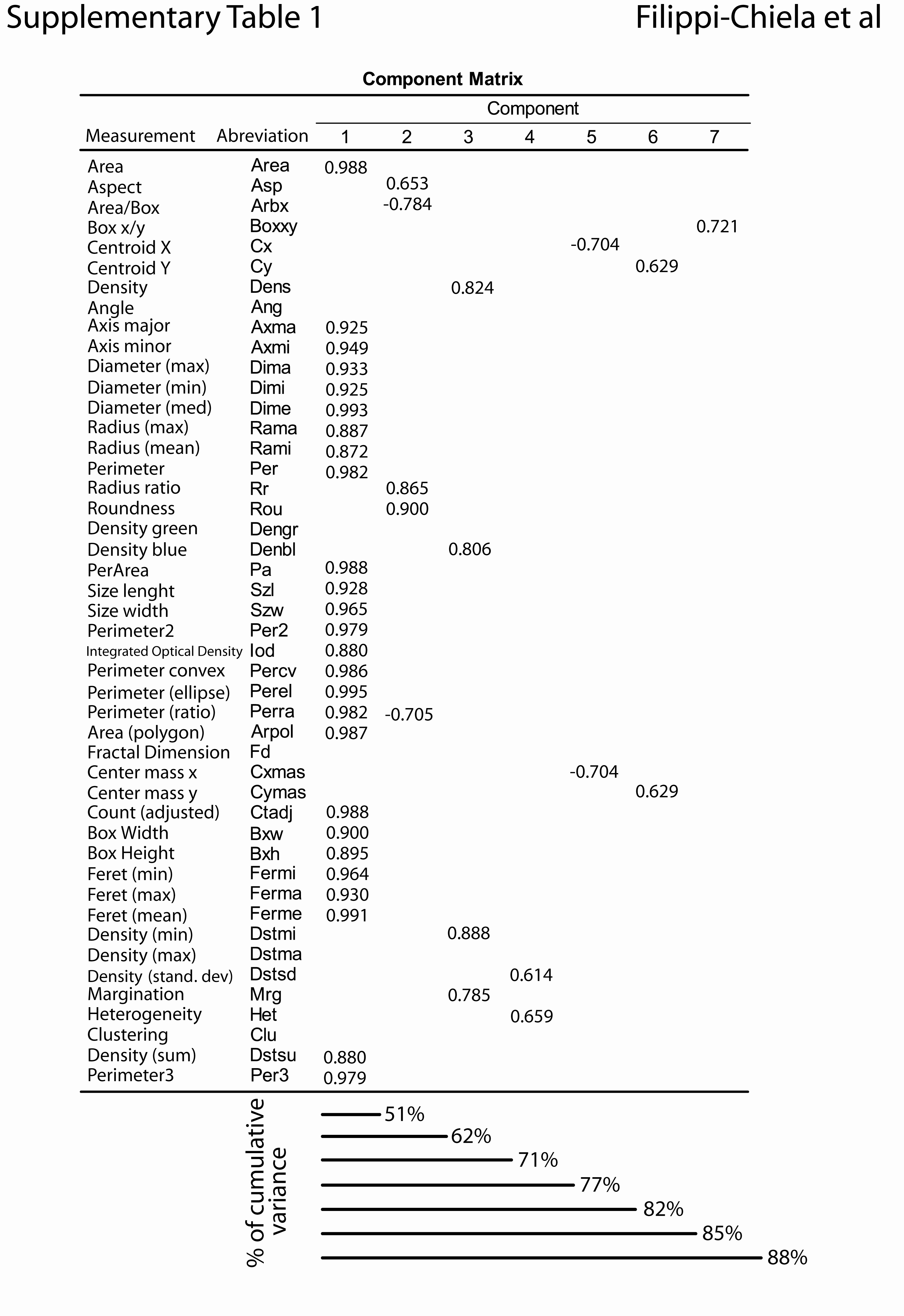


**Supp. Results Table 1 – Principal component analysis (PCA) of morphometry measurements obtained using Image Pro Plus 6.0 (IPP6).** Values represent the correlation of the variable with the correspondent principal component (PC). In the bottom of the table the cumulative percentage of the total variability of represented by the PC is shown.

**Statistical method for discovering the measurements that better separate populations based on nuclear morphology**

We initially grouped the nuclei based on typical morphology, as N (Normal), M (Mitotic), L (Large) and Ir (Irregular). The nuclei clearly presented characteristics of these phenotypes **(Supp. Results Fig. 1)**. The measurements of the 46 variables underwent Principal Component Analysis (PCA) using the software PASW Statistics 18 (formerly known as SPSS). The first Principal Component (PC1) accounted for 51% of the total variability and presented high (> 0.87) correlation values with measurements directly related to size (area, perimeter, diameter and so forth, in a total of 26 major measurements) **(Supp. Results Table 1**).

Measurements of shape and regularity such as aspect, area/box, roundness and radius ratio had absolute correlations higher than 0.65 with PC2, which accounted for additional 11% of total variability. These two components together represented 62% of the variance. A successful separation of N, M, L and Ir nuclei were obtained plotting PC1 versus PC2 values. Confirming the characteristic of size of PC1, a plot of area versus PC2 was as efficient in separating these groups of nuclei as was PC1 versus PC2 **(Supp. Results Fig. 1)**. This suggests that area alone is sufficient to represent the variability represented by PC1. Plots of PC3 to PC5 versus area or PC2 did not produce a good separation of the groups *(data not shown)*.


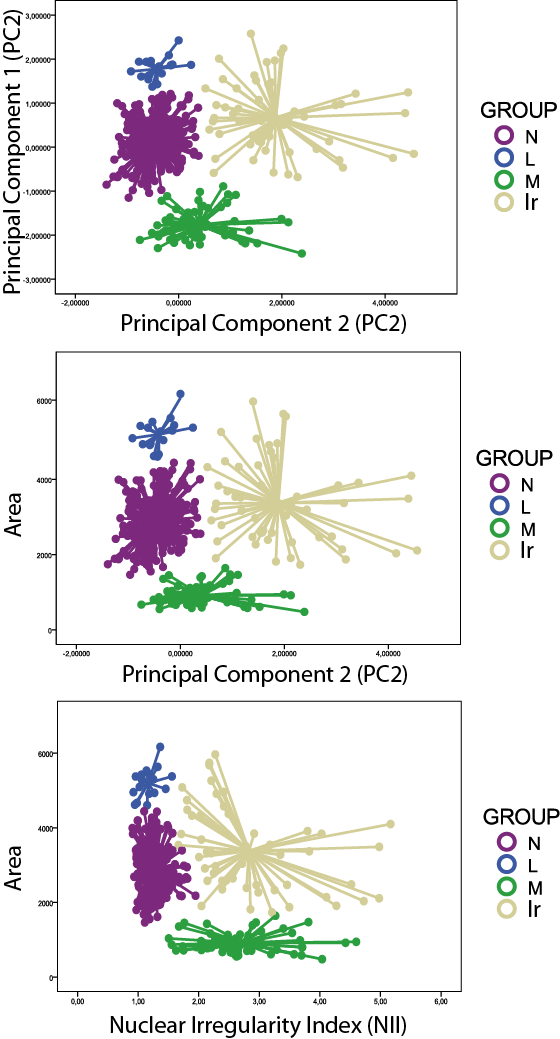


**Supp. Results Figure 1 – Separation of nuclei using data from Principal Component Analysis (PCA).** Separation was performed using PC1 and PC2 (top), Area and PC2 (mid), Area and NII (bottom). N=normal; L=Large; M=mitotic; Ir=irregular.

In order to assess the importance of each of the five more influential measurements on PC2, we used a multivariate analysis of variance (MANOVA) with Student-Neuman-Keuls post-hoc test to compared N, L, M and Ir nuclei in relation to these variables. None of the five variables of PC2 (Aspect, Area/Box, Radius Ratio, Roundness, Perimeter Ratio) efficiently separated N and L nuclei, since these populations are distinguished by Area (or PC1) and have similar PC2 values **(Supp. Results Fig. 1)**. On the other hand, all measurements alone efficiently discriminated groups N and L from M and Ir, except perimeter ratio, which did not separate N and L from M nuclei **(Supp. Results Fig. 2**). Therefore, Perimeter Ratio was dropped from the analysis.


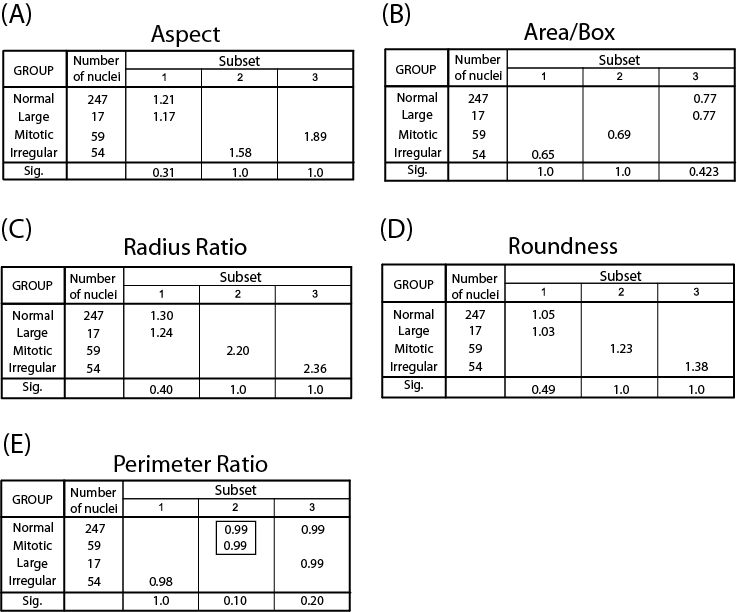


**Figure 2 – Discrimination of variables that compound PC2 in separate groups of nuclei.** Variables were tested for its capacity to separate nuclear population. As shown, only Perimeter Ratio was not able to separate populations of nuclei N and M – as marked in (E), as performed by PC2 (see Supp. Results Figure 1).

Finally, the variables that better described regularity - Aspect (Asp), AreaBox (Arbx), Radius ratio (Rr) and Roundness (Rou) - were combined in an index called Nuclear Irregularity Index (NII),. In all PCAs, Arbx presented a negative correlation, which is understandable since more complex objects have a lower Arbx value. Therefore, the NII is given by:

NII = Asp-Arbx+Rr+Rou

A graph of Area versus NII was very similar to PC1 versus PC2, (**Supp. Results Fig. 1 – bottom)** showing that the variable reduction strategy (from 46 to five measurements) adopted did not significantly reduce the description of the important aspects of nuclear morphology. It is important to notice that these weights were established based on our populations of nuclei and that data from other cell types or treatments may require other weights. Therefore these weights are changeable in the Excel file available to users.

**Development and Validation of a Plugin for the Morphometric Analysis using the free Software Image J**

In order to permit a wider use of this tool, we developed an Image J plugin that allows the quantification of the five measurements that are part of the morphometric analysis. Similarly to IPP6, images of nuclei stained with DAPI are necessary to do the nuclear analysis using the Image J, with 300 dpi or more to guarantee the quality of the results (for more details about the Plugin and a user manual, see *Supp. Methods file*).

The mathematics and the objective of the Plugin is very similar to the analysis using IPP6, with some improvements, since it was developed specifically for this task. As soon as the plugin starts to execute, it will outline all the nuclei found for the current parameters set. As for IPP6, new nuclei can appear/disappear if the parameters are changed, or can be manually inserted by the user.

The individual values obtained with IPP6 and Image J for the five measurements as well as for NII are presented for seven examples of representative nuclei **(Supp Results Fig. 3)**. A comparison of data generated by IPP6 and our Image J plugin did not differ in more than 85% for all measurements and for NII in a population of more than 50 nuclei.


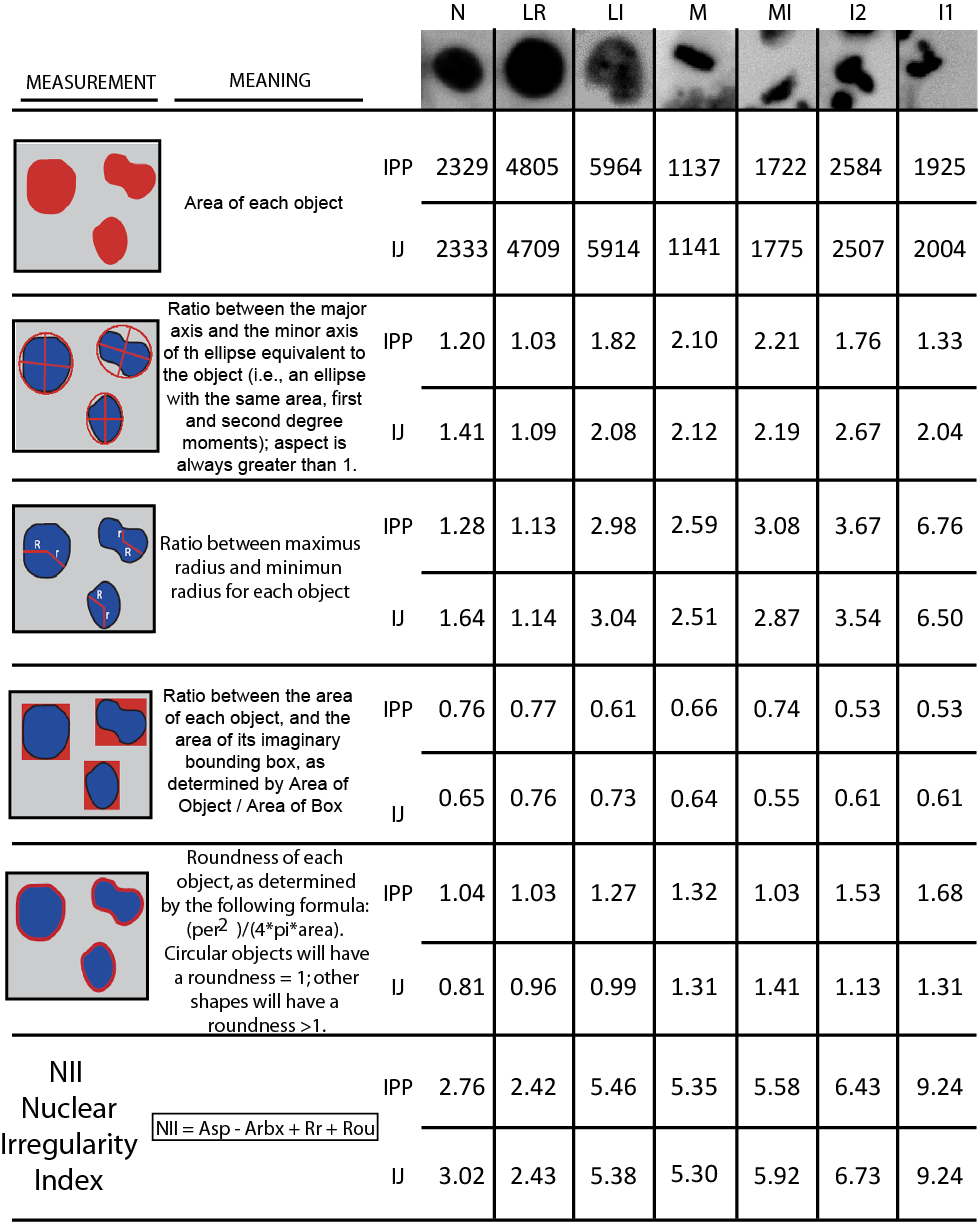


**Supp. Results Figure 3 – Illustration and comparison between IPP6 and ImageJ of measurements that best represent the variability observed in nuclear morphology.** Graphical representation and meaning of morphometric variables given by IPP6 software as well as measurements from seven representative nuclei that belong to the features used to evaluate the separation between groups using IPP6 and Image J (IJ).

Further, comparisons of the percentage of nuclei in each population using IPP6 and ImageJ confirmed the validation of the ImageJ plugin developed to perform NMA **(Supp. Results Fig. 4).**


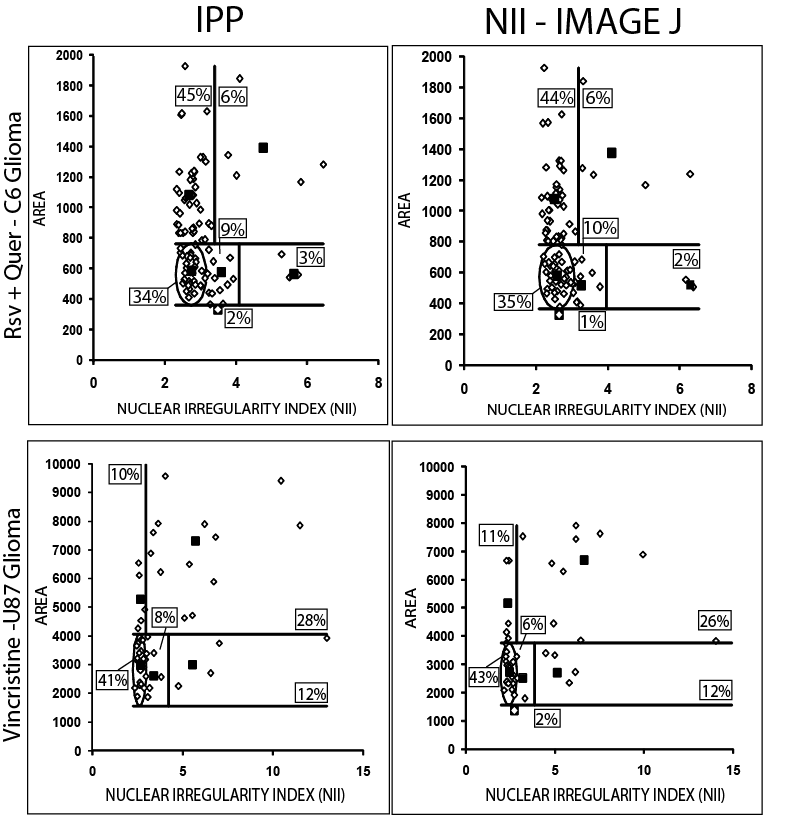


**Supp. Results Fig. 4 - Graphs comparing nuclear separation using IPP6 and Image J data**. Numbers in boxes denote the percentage of nuclei in each category. MC – mitotic catastrophe; NII-Image-J – Nuclear Index Irregularity using the plugin to Image J

**Development of the file to analyze raw data**

Next we set up a user friendly Excel file to allow easy analysis of nuclear morphometric data. The first spreadsheet (named ‘Normal nuclei and Settings’) is where normal nuclei data (area, aspect, area box, radius ratio and roundness from nuclei with a regular/normal shape) are inserted. NII is automatically calculated based on the relative weights applied to each measurement. The averaged area and NII of these measurements are calculated, followed by generating an elliptic plot, here named ‘normal ellipse’, obtained considering the number of Standard Deviations (SD) from the mean. The number of SD to define the ‘normal ellipse’ must be changed by the user, so that the line circulates the population of nuclei (to see examples, see *Supporting Methods file*). The ‘normal ellipse’ will be the reference data for the other spreadsheets.

Still in the first spreadsheet, a place to define the “settings” of the analysis is available to the user. User must paste all data obtained in the yellow box indicated and set the number of standard deviations to separate the populations of nuclei, similarly to a flow cytometric analysis. Examples of this procedure can be observed in the *Supporting Methods file.* Many spreadsheets (named as *“Test”*) may be include that will contain control and treated nuclei. The user may increase the number of spreadsheets, dragging any ‘Test’ spreadsheet to the right while pressing the *Ctrl* key (in windows). Inserting the data from the tests will produce a plot of area versus NII to each condition. Furthermore, averaged area and NII of each population of nuclei, beyond SD of these variables, are calculated to the experiments. The average of each population is plotted in the graphs **(Supp. Results Fig. 4).**
