# Supplementary material for: Nuclear Morphometric Analysis (NMA): Screening of Senescence, Apoptosis and Nuclear Irregularities
Source: PLoS One. 2012 Aug 8;7(8):e42522. doi: 10.1371/journal.pone.0042522 (PMC3414464; doi:10.1371/journal.pone.0042522)
Supplement: Text S2 — Supporting Methods. This file describes the instructions of using Image J, IPP and Spreadsheets to perform the NMA analysis. (DOC) [file pone.0042522.s006.doc]

**Nuclear Morphometric Analysis (NMA):**

**Screening of apoptosis, mitosis, senescence and mitotic catastrophe**

Eduardo C. Filippi-Chiela^1^, Manuel M. Oliveira^3^, Bruno Jurkovski^3^, Sidia Maria Callegari-Jacques^4^, Vinicius Duval da Silva^5^; Guido Lenz^1,2^,*

# Supporting Methods

**_________________________**

# This supporting file describes the instructions of using Image J, IPP and Spreadsheets to perform the NMA analysis.

1. Image J Plugin Manual
2. Image Pro-Plus Manual
3. Spreadsheet manual

# A. Image J Plugin Manual

# 1 Introduction

The NII Plugin of the Image J Software was developed to extract the morphometric information about nuclei to be used in the NMA tool. The analysis uses measures of nuclear area and of four parameters of irregularity, named Aspect, Area/Box, Radius Ratio and Roundness. These four parameters are used to generate a Nuclear Irregularity Index (NII) which, added to area measurement, classify the nuclei in normal (N), irregular (I), small and regular (SR), small (S), small and irregular (SI), large and regular) or large and irregular (LR)

Images of nuclei stained with DAPI (with more than 300 dpi; preferentially in .tiff uncompressed format) are necessary to guarantee the quality of the nuclear analysis. This manual describes the steps for using the NII Plugin in the NMA.

# 2 First Steps

## 2.1 Getting Image J

Install ImageJ (version 1.4.4), (<http://rsbweb.nih.gov/ij/>) and NII plugin ([www.ufrgs.br/labsinal/NMA](http://www.ufrgs.br/labsinal/NMA)).

## 2.2 Installing NII Plugin

After installing ImageJ, create a new folder named NII, into ImageJ’s plugin folder, and simple paste the NII_Plugin to this folder. The next time ImageJ is opened, the plugin will appear in its plugin list **(Figure 1).**

Figure 1. Instaling NII_plugin

## 2.3 Running the plugin

Open a.TIFF image by choosing Open in the File menu (or pressing *Ctrl+O*) in ImageJ. Then just choose NII_Plugin in the Plugins menu opens a setting part on the right of the figure **(Figure 2)** and automatically outlines the nuclei.

#
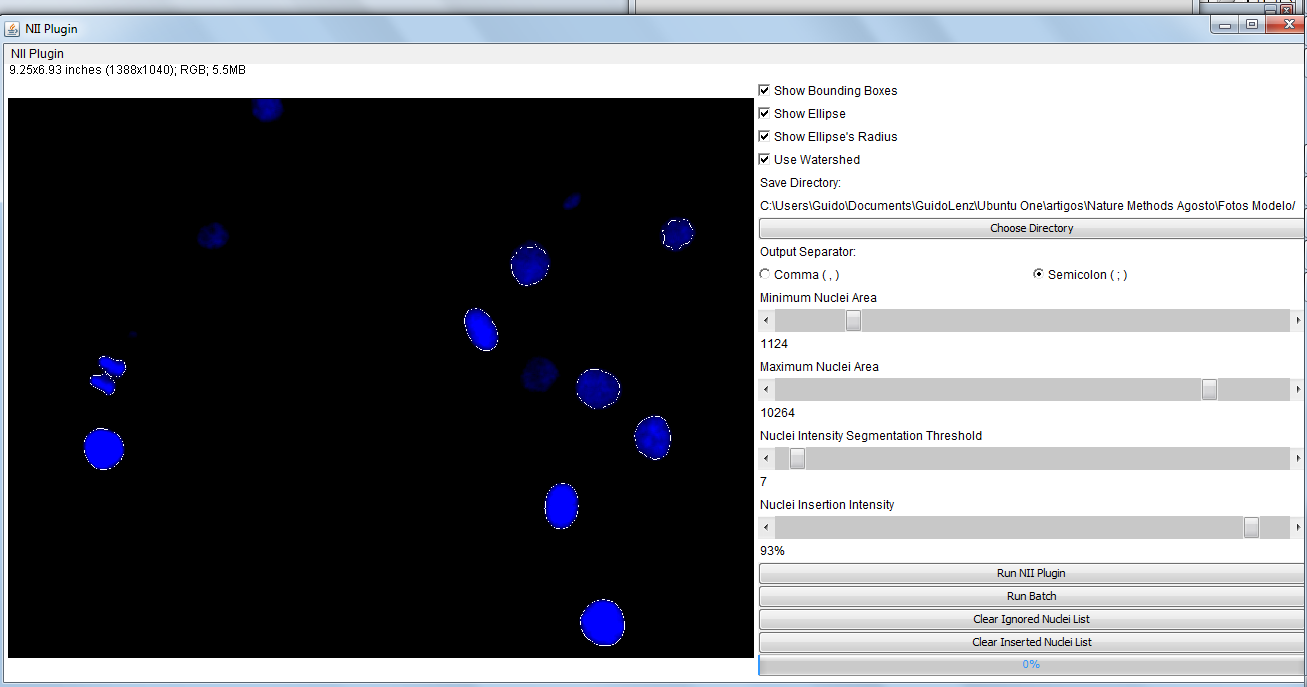


Figure 2. Opening NII_plugin with the options for the image analysis.

# 3 The Plugin

This section presents the characteristics of the plugin specifically designed to NMA using ImageJ. Next section (*section 4 – “Analyzing”*) describes the steps to execute NMA using the plugin.

## 3.1 Running Parameters

- **Choose Directory** in which the outputs will be saved.
- **Use Watershed:** if this option is checked, the plugin will try to separate some of the nuclei that are touching each other. It may, however, improperly separate a very irregular nucleus, so the user has to take care when using this option (mainly for batch processing).
- **Minimum Nuclei Area:** the minimum area (in pixels) of a nucleus to be considered by the program.
- **Maximum Nuclei Area:** the maximum area (in pixels) of a nucleus in order to be considered.
- **Nuclei Intensity Segmentation Threshold:** this is the first option that is altered to modulate the sensitivity of marking and to adjust the nuclear surrounding line in the correct limit (*i.e.* the threshold used to discretize the grayscale image to a binary one). The smaller the threshold, less intense nucleus will be considered in the analysis (and the greater will be its area).
- **Nuclei Insertion Intensity:** this is the percentage of the Segmentation Threshold used when inserting a blob manually.

## 3.2 Adding and Removing a Single Nuclei Manually

General marking of nuclei is obtained by using the ‘Nuclei Intensity Segmentation Threshold’, as described above. To add a nucleus that wasn’t detected by the program, just click inside its area with the Left Button of the mouse while pressing the ***Shift* key** and for removing a selected nuclei, hold the ***Ctrl* key** (for Mac users, use the 64 bits image J and the control key to remove and shift key to include nuclei). If necessary, it’s possible to remove all inserted nuclei or re-insert all removed nuclei just clicking the Clear Inserted Nuclei List or Clear Ignored Nuclei List buttons.

## 3.3 The Output

After running the plugin for an image, three files will be generated: 1. copy of the original image with the ID of the considered blobs and the additional information selected by the user in the interface *(section 3.4, below)*; 2. a binary image containing only the blobs that were processed by the plugin. Both will be saved as a .png ﬁle which will be suﬁxed with _NII and _NII_Binary; 3. a Comma Separated Values (.csv) ﬁle that can be opened with any program that manipulates spreadsheets, such as Microsoft Office Excel or OpenOffice Calc. The user can choose between a comma (,) or a semicolon (;) separator in the interface depending on the settings of the computer. The values stored for each nucleus in the table are the components of the Nuclear Irregularity Index (NII), as follows:

- **Area:** area of each nucleus.
- **Aspect:** ratio between the nucleus’ major and minor radius.
- **Area/Box:** ratio between the area of the nucleus and the area of its bounding box.
- **Radius Ratio:** ratio between the maximum and the minimum radius of the object.
- **Roundness (Border):** roundness computed using the Border Perimeter[[1]](#footnote-2). It is determined by the formula: perimeter²/(4∗*pi*∗area), *pi* being the mathematical constant.

These variables are used to yield the NII, and are the fundamental measurements of NMA. They are based on the following information, obtained for each nuclei after running the NMA plugin:

- **Roundness (Ellipse):** roundness computed using the Ellipse Perimeter, with the same formula of the variable named *roundness (border)*, cited above.
- **Bounding Box Area:** area of the smallest box that can wrap the nucleus.
- **Centroid Coordinates:** (x,y) coordinates of the nucleus center of mass.
- **Minimum Radius:** minimum distance from the centroid to a border of the nucleus.
- **Maximum Radius:** maximum distance from the centroid to a border of the nucleus.
- **Perimeter (Border):** perimeter measure computed using the nucleus border.
- **Perimeter (Ellipse):** perimeter measure computed using the ellipse’s perimeter formula.
- **Aspect Equivalent Ellipse:** similar to Aspect but uses the radius of an ellipse with the same area as the nucleus.
- **The parameters used in the computation of data:** minimum nuclei area, maximum nuclei area, nuclei intensity segmentation threshold (described in 3.1).

## 3.4 Output Options (exemplified in section 4)

- **Show Bounding Boxes:** shows the bounding boxes of each nucleus in the output images.
- **Show Ellipse:** shows the ellipses that circulate each nucleus in the output images.
- **Show Ellipse’s Radius:** shows the ellipse’s radius of each nucleus in the output images.
- **Save Directory:** the directory in which the output ﬁles will be created.
- **Output Separator:** the character used to separate values in the generated table.

## 3.5 Conﬁguration File

The ﬁrst time the plugin is executed, it will generates a niiConﬁg.txt ﬁle in the NII_Plugin folder. This ﬁle contains some parameters of the plugin, and can be changed if necessary. These parameters are:

- **minArea.maxValue**: the minimum value that the Minimum Nuclei Area scrollbar can have.
- **maxArea.maxValue:** the maximum value that the Maximum Nuclei Area scrollbar can have.
- **window.width:** the plugin window width.
- **window.height:** the plugin window height.

If for some reason the ﬁle gets corrupted or if you want to restore its default values, just remove it from the NII_Plugin folder the next time the NII Plugin executes it will create the original ﬁle again.

## 3.6 Batch Processing

To run the plugin for a batch of ﬁles, the user is prompted to define in which directory the input ﬁles are. The plugin will then run, with the same characteristics, for every image contained in that directory. The valid image formats are: tif (preferable but not restrict to), jpg, jpeg, bmp, png, gif. Like the execution for a single ﬁle, the outputs will be saved in the directory specified by the Save Directory option.

# 4 Analyzing

## 4.1Analyzing the nuclei

After opening your file on Image J (tab *file* – *open)*, select the *NII Plugin* on plugins tab, followed by these steps:

**i. Adjust** nuclear marking using the *Nuclei Intensity Segmentation Threshold*, until the line that limits the object surrounds it perfectly **(Figure 3)**.

**Caution!** Take care to avoid overmarking or mismarking. Selecting the option “Use watershed” the user reduces the juncture of two very close nuclei **(Figure 3c-d)**.

**Caution!** Also to avoid mismarking of overlapping nuclei and/or the marking of small unspecific objects, use the tools *Minimun* and *Maximun Nuclei Area* to establish an interval of area to be analyzed.

**ii.** After the group marking of nuclei, an analysis of the image must be done. Exclude mismarked nuclei pressing the *Ctrl* key and clicking into the nucleus (**Figure 3d, white arrows**). To include a non-marked nucleus, press the *Shift* key and click into the nucleus **(Figure 3f, white double arrows**). Adjust the intensity of these inserted nuclei using the option *insertion intensity option* of the plugin **(Figure 3f).** Inserted and excluded nuclei can be ignored through the options *clear ignored nuclei list* and *clear inserted nuclei list*, respectively.


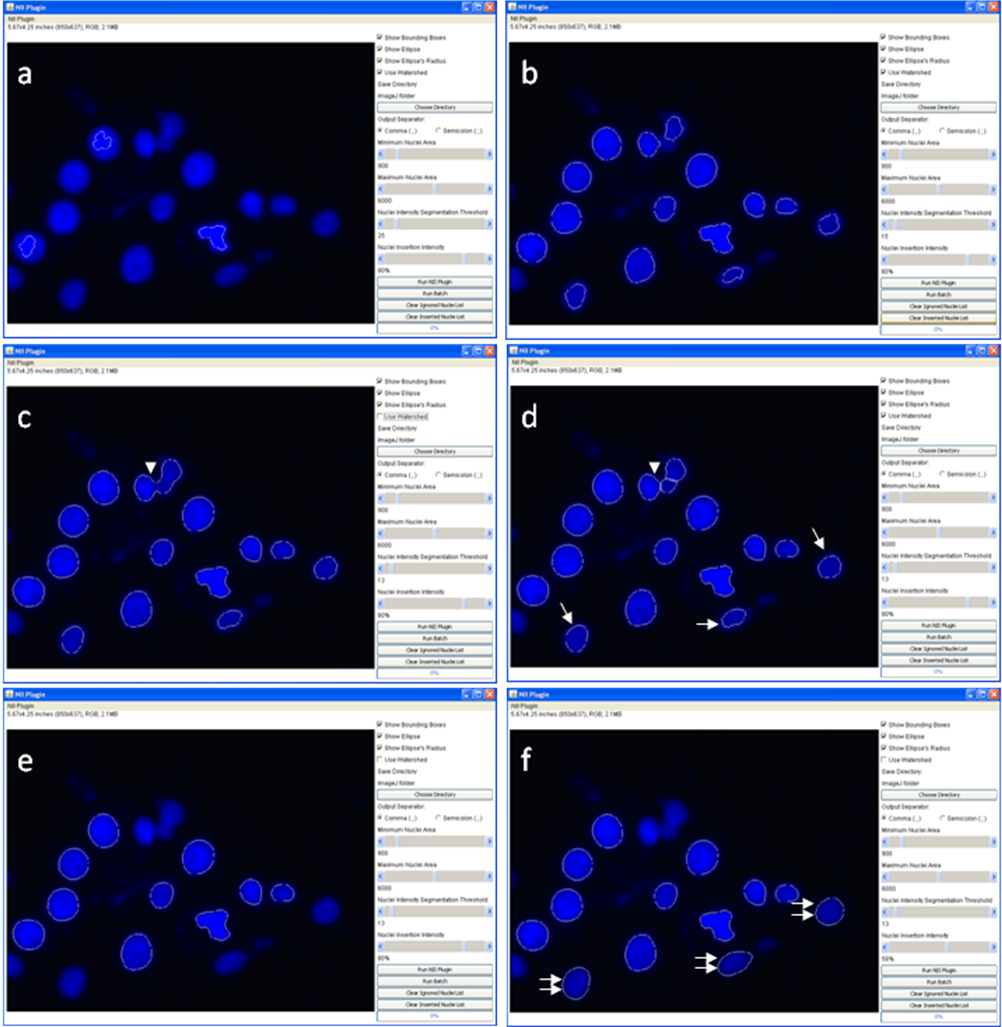


Figure 3. Nuclear marking using NII plugin in Image J. (a-c) Nuclei being marked gradually until the correct limit. (c-d) Watershed option (not selected in c and selected in d) helps to separate two nuclei very close, as pointed by the white arrowhead in both images. As indicated by single arrows (d), some nuclei are mismarked and must be remarked. The user can exclude these nuclei pressing C*trl* key and clicking on them (e), followed by re-inserting these nuclei individually, by clicking individually in each of them and adjusting the intensity of marking through the nuclei *insertion intensity option* of plugin (f – double arrows). It is important to note that some nuclei cannot be available to measuring in a given image, such as those marked with an arrowhead in letter d. To see details of the plugin window, see Figure 4.

**ii.** Select the appropriate **output options**.

It is important to determine the output separator, if comma or semicolon, and select the options of the output file, as described on 3.2, which will help the user to localize and evaluate data after acquisition. The user can also choose the directory where output data will be placed. All output files are generated with the name of the original image analyzed.

**iii.** After adjusting the outline of the largest possible number of nuclei, **run NII plugin** and wait until the bar reaches one hundred percent **(Figure 4).**


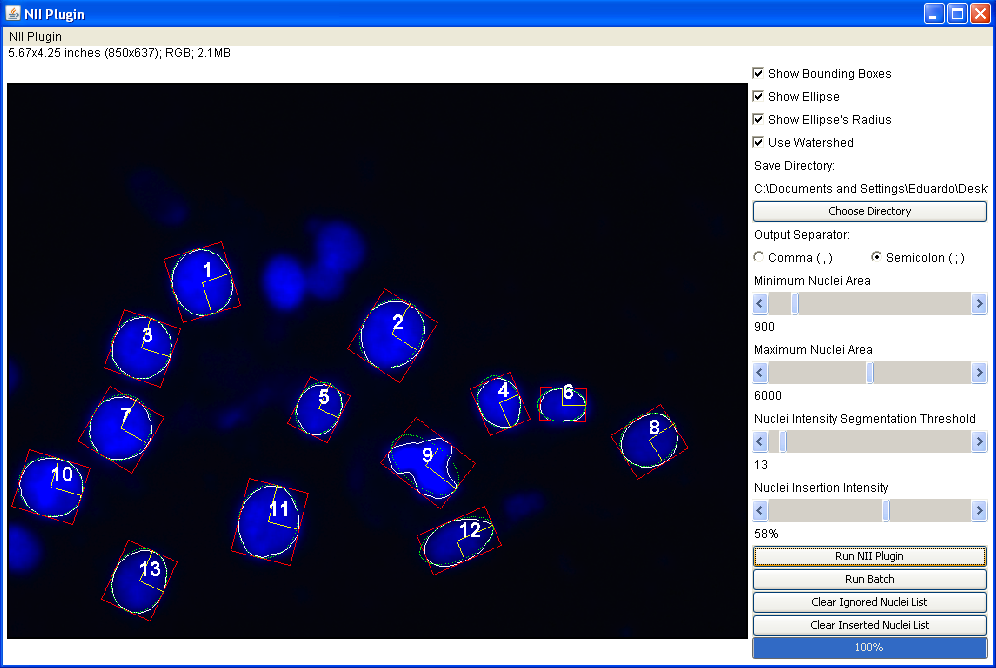


Figure 4. Image after running NII plugin. After marking and running the plugin, nuclei are marked with the bounding boxes (red), ellipse (green) and radio of the ellipse (white lines into nuclei), if these options had been selected by the user in the bottom right options of the plugin window.

**CAUTION**! If executing a Batch analysis, the user must check each image generated to avoid mismarks.

**iv.** After running NII, three files are generated to each image. Open the **Excel file (Figure 5A)** and copy data obtained to Area, Aspect, Area Box, Radius Ratio and Roundness to the Excel file, in the appropriate place. To examine individually a nuclei, open the output images **(Figure 5B, C)**.


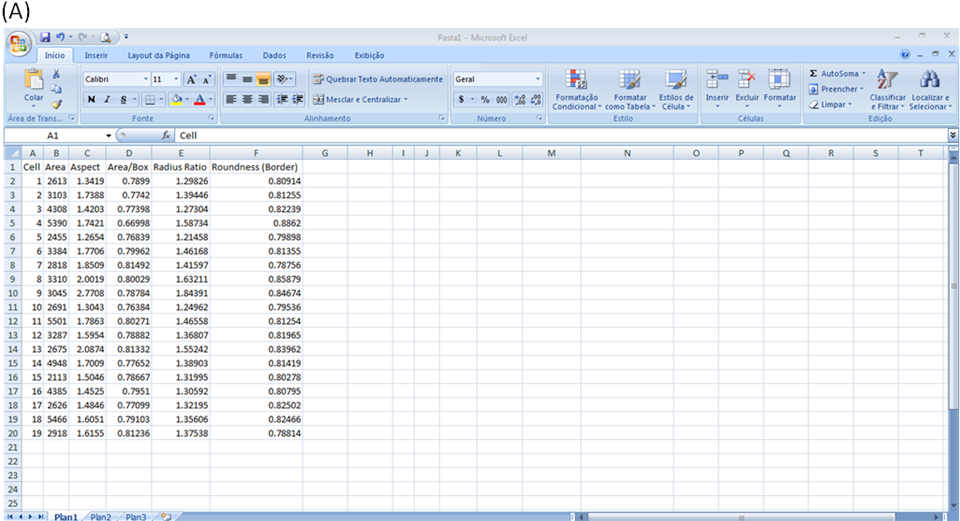


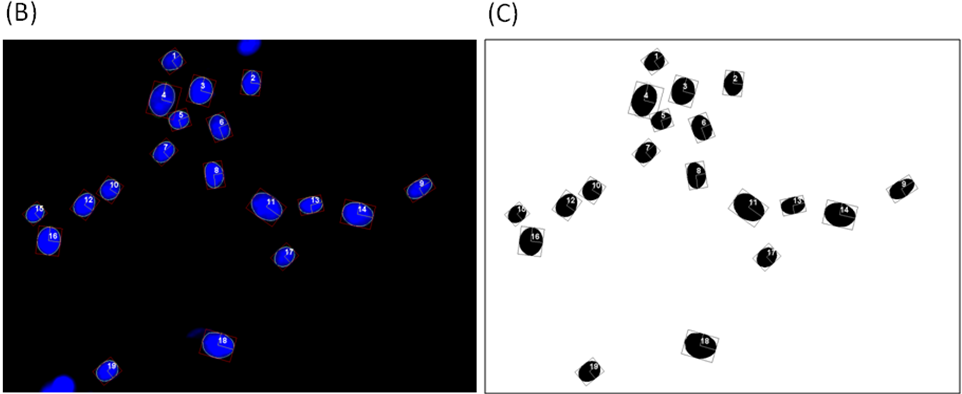


Figure 5. Output data from NII plugin. (A) Excel file gives data of Area, Aspect, Area/Box, Radius Ratio and Roundness of each cell; (B) image of nuclei with marking as in Image J Software; (C) binary file of the analyzed image.

# B. Image Pro-Plus 6.0 (IPP) Manual

**1.** Open your tiff image on IPP

**2.** In the tab *measure* select *count/size*. Into the count/size window, select the tab *measure*, followed by *select measurements.* In the new window opened, click on the measurements Area, Area/Box, Aspect, Radius Ratio and Roundness, and click *ok*.

The selection of nuclei can be done individually or for all nuclei together. The next sections present both options.

**A. GROUP SELECTION OF NUCLEI**

**1.** To select nuclei, in the *count/size* window select the option *select colors.* Into the new opened window (named *segmentation)*, select the option 1x1 in the scale field (
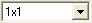
). After that, select the icon
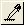
 and click continuously onto a nucleus of the photo with the left mouse button until the marking reaches the correct limit of nuclei **(Figure 6Aa-e)**. Use the icons
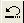
,
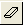
 and
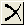
, respectively, to go back a click, to reduce the marking gradually or to undo the whole marking and start again.

**2.** After getting a correct nuclear marking, *close* the *segmentation* window. At this moment, nuclear marking will disappear (don’t worry!). Click on *count* and nuclei become marked as defined **(Figure 4Af)**. Mismarked nuclei can be eliminated by clicking on *view* tab and, after, *object window*. In the *object attributes* window, select to *hide* those mismarked nuclei.

**CAUTION!** Take care that you do not overmark or mismark nuclei, which may generate unreal data, as exemplified on **Figure 6B**.


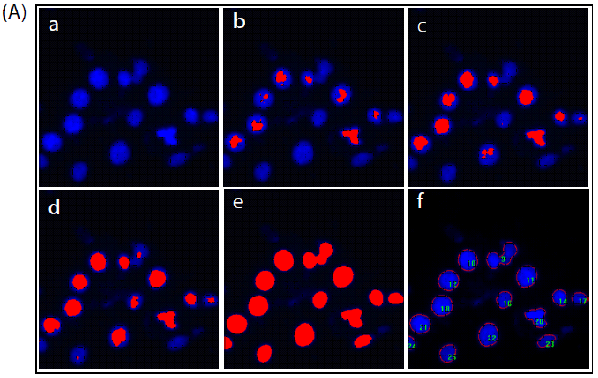


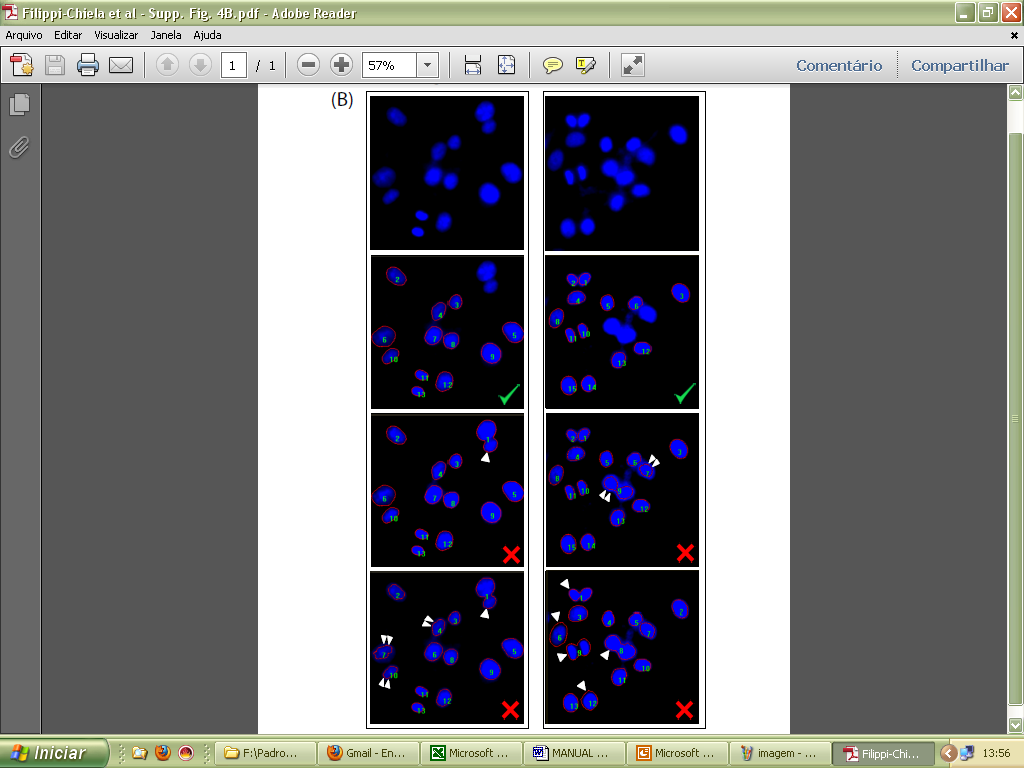


Figure 5. Examples of group nuclear selection in IPP. (A) Gradual nuclei marking using IPP through the *Group Selection* method described on the text. (a-e) show the gradual marking of nuclei, while (f) shows the limit of each object marked after running the analysis. Nuclei grouped in object 9 are mismarked (see a), and must be eliminated from the analysis. (B) shows two examples of correct (green correct sign) and incorrect (red ‘x’ sign) nuclei marking. Single arrowheads illustrate nuclei that are overmarked, while double arrowheads show insufficient marked nuclei. All these cases must be eliminated from the analysis.

**3.** To get the output data, go to *file* tab and select *data to clipboard*. Open an Excel file and paste your raw data, followed by copy and paste measurement data to **NII Excel file**.

**B. INDIVIDUAL SELECTION OF NUCLEI**

**1.** After opening the image and selecting the measurements, select this icon
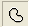
 in the toolbar and the option *wand* of the new opened box. Click in a single nucleus with the left mouse button until the correct marking **(Figure 7a-b)**. When the nucleus is correctly marked, click on another nuclei while pressing the *Ctrl* key. The marked nucleus becomes green and the new nucleus to be marked appears in red **(Figure 7b)**. Proceed as done for the first nuclei and follow these steps until all nuclei are green marked **(Figure 7c)**.

**2.** After marking as many nuclei as possible, select the tab *measure*, in the count/size window, followed by *select measurements;* in the new window opened, click on the measurements Area, Area/Box, Aspect, Radius Ratio and Roundness to select them, and click *ok.*

**3.** In the *count/size* window select the option *convert AOI to object(s)*, in the *Edit* tab; nuclei will become numbered as in the last step of group analysis **(Figure 7d)**.


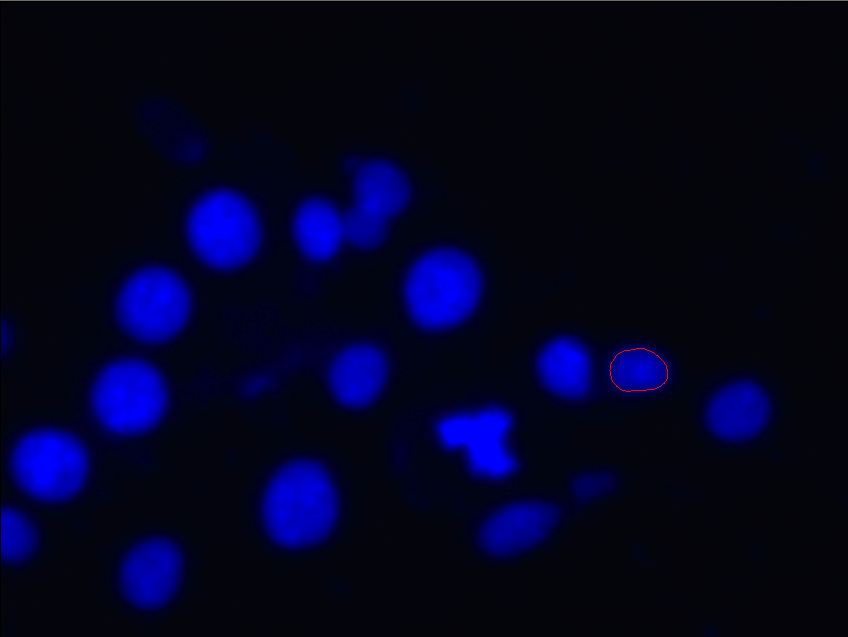

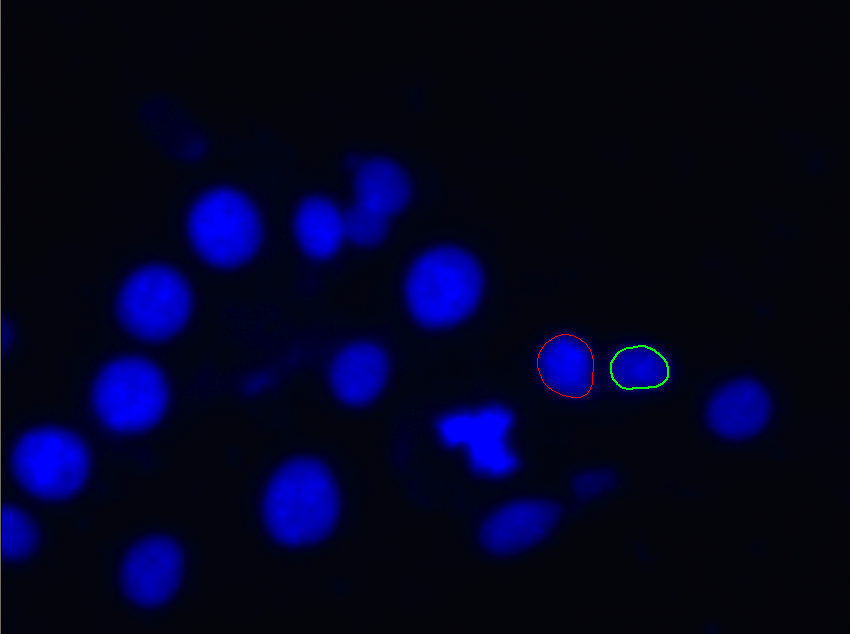

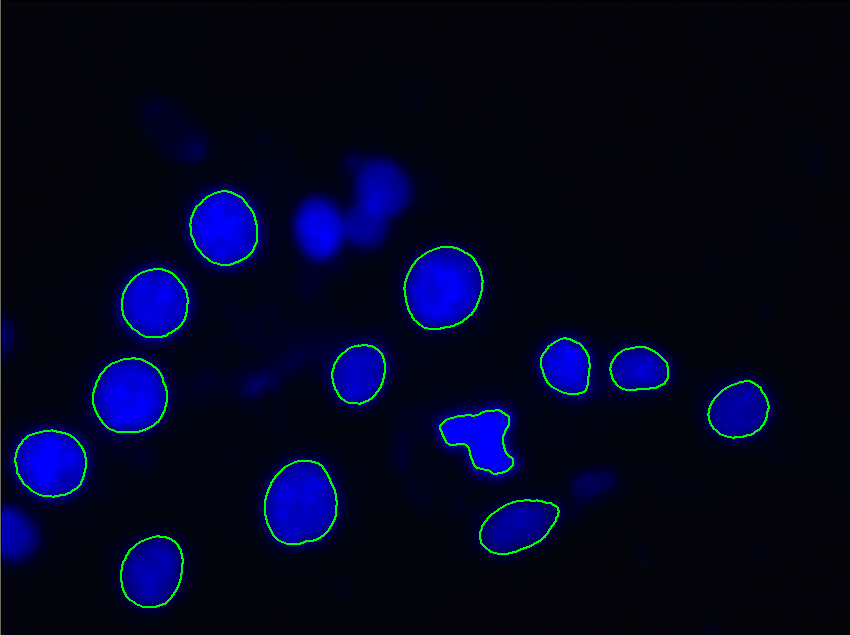

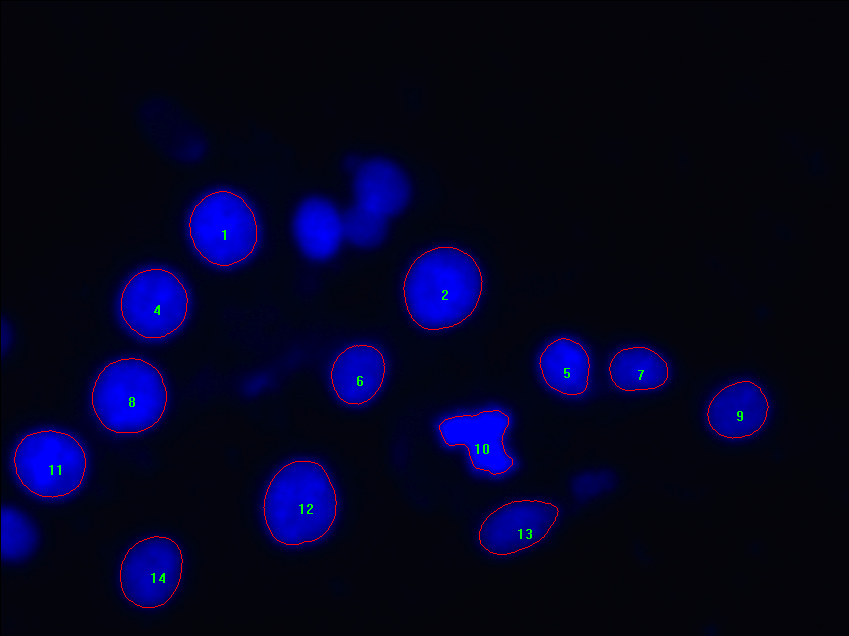


a

b

c

d

Figure 6. Individual marking using IPP. After opening the image, a nucleus is marked individually (a), selected (b) and, when all nuclei are selected (c), they are converted to objects (d) and output data produced.

**CAUTION!** As in the group analysis, check that you do not overmark or mismark nuclei, which may generate unreal data.

**4.** To extract output data, go to *file* tab and select *data to clipboard*. Then open an Excel file and paste your raw data, followed by copy and paste measurement data to **NII Excel file**.

# C. Spreadsheet Manual

**1.** Use different files for different cell type of your test, since different cells have different nuclear features and the data from the “Normal Nuclei and Settings” will apply for the whole file

2. Create one spreadsheet for each condition of your test (treatments, time of analysis, etc). To do this, select the spreadsheet named “**Condition1”,** press *Ctrl* and slither the spreadsheet aside. Rename the new spreadsheets formed according to the treatment. Data from control must be allocated in the spreadsheet named “**Control**”.

**2.** Paste your data from normal nuclei, obtained in IPP6 or ImageJ Plugin, to the related columns of “NORMAL NUCLEI” area (C11-G11) in the spreadsheet named **“Normal Nuclei and Settings”.**

**CAUTION!** Have in mind that “normal nuclei” do not refer to nuclei with a round shape. Many cell types present, even when healthy, a high variety of nuclei shapes. The user needs to choose a group of nuclei that represent this variability of basal cell culture condition. However, nuclei from mitotic cells and nuclei with clear abnormalities should be eliminated from this section.

**3.** Afterwards, if using Excel, exclude the formula of NII (orange column – column H) of those lines without data.

**CAUTION!** Forgetting the formula in lines without raw data (as X will appear) hampers not only the formation of the ‘normal ellipse’ (**Figure 8**), but also the analysis of the experimental data (Figure 8). Do not forget to delete it! In OpenOffice it is unnecessary.


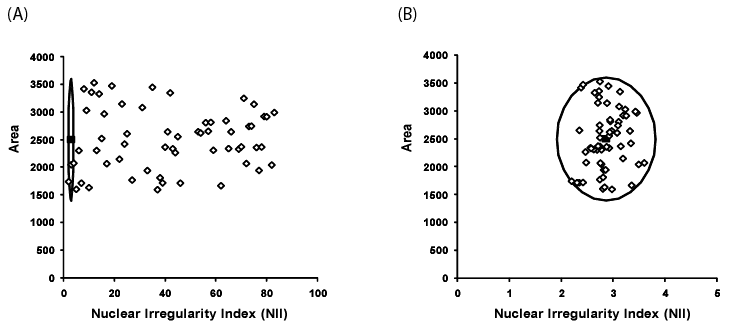


Figure 7. Results of ‘normal ellipse’ determination. **(A) Wrong distribution and ‘normal ellipse’ formation when the formula of NII is maintained in the lines where there is no raw data (as X will appear); (B) Correct ‘normal ellipse’ formation after deletion of NII formula from those lines without data to calculate NII.**

**4.** Observe the distribution of nuclei in the graph. The distribution must be homogeneous and uniform. If a nucleus is located far outside the ellipse, delete it, since probably it is not a normal nucleus. The user can modify the number of standard deviations (SD) of Area and NII (in the option “How many SD is N” of the box “SETTING THE NORMAL POPULATION” – green cells (P2 and P3 – standard is 2) so that the ellipse is formed as representative as possible around the distribution of the normal nuclei.

**CAUTION!** To improve the determination of the ‘normal ellipse’, change the number of SD. Excessive or low number of standard deviations lead to the formation of a non-representative ellipse, as exemplified in **Figure 9.**


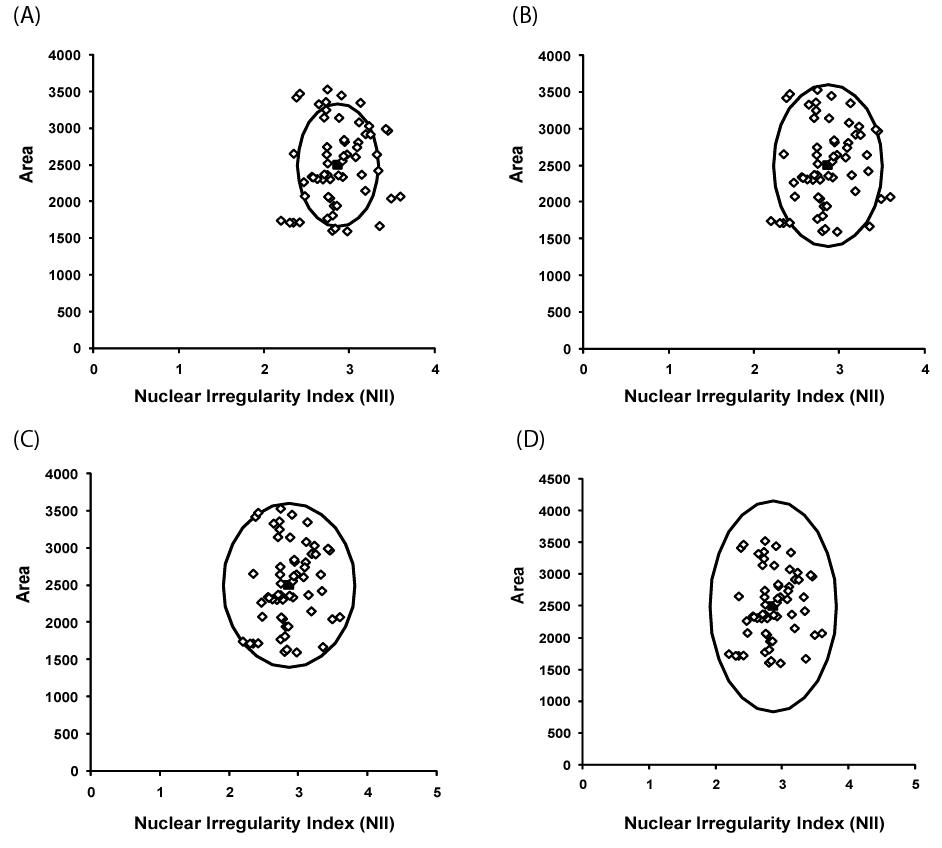


Figure 8. Determination of the ‘normal ellipse’. **(A) Very small value on the settings (P2, P3) (B) A value slightly to low; nuclei that are out the ellipse could be deleted to improve the homogeneity of the group of normal nuclei; (C) correct value; (D) Value to high.**

**5**. Paste all analyzed data (Area, Aspect, Area/Box, Radius Ratio and Roundness - from all conditions) in the respective columns of “TREATED NUCLEI (FOR SETTING)” area (Z11-AD11), in the spreadsheet **“Normal Nuclei and Settings”.**

**CAUTION!** Forgetting the formula of NII in lines without raw data hampers the analysis of the experimental data **(Figure 10).** Do not forget to delete the Xs if using Excel!


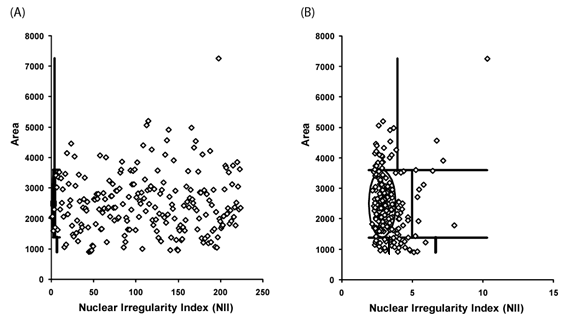


Figure 9. Results of separation of nuclei to determine the appropriated settings to the analysis. **(A) Wrong distribution when the formula of NII is maintained in the lines where there is no raw data; (B) Correct separation of populations after deletion of NII formula from those lines without data to calculate NII.**

**6.** Adjust the number of SD that divides populations of nuclei in the options “How many SD is LI, SI and I” of the box “SETTING OF ALL OTHER POPULATIONS” – green cells. Take care to have a representative number of nuclei in each population and the correct separation of them.

**CAUTION!** This step is fundamental in the NMA. Use as much nuclei you have, from all your treatments, so that you can define the best thresholds for the different populations. As exemplified in **Figure 9,** a wrong setting will divide the populations of nuclei nonspecifically, and the analysis will be incorrect.


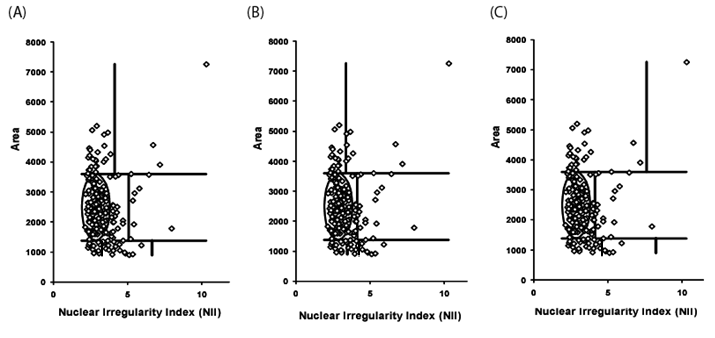


Figure 10. Determination of the settings to separate the populations of nuclei. **(A) Correct setting to separate the populations of N, I, LR, LI, SR, S and SI; (B) Settings to separate LR of LI, N of I, and S of SI are too low; (C) Setting to separate LR of LI, SR of S and S of SI are too high.**

**6.** Paste the data obtained for nuclei of cells exposed to the different conditions in the related spreadsheet.

**CAUTION!** Each experiment must be pasted in the appropriate location.The plugin provides boxes for a total of ten experiments, with a place for 500 nuclei for each condition (Experiment 1 from line 13 to 512, Experiment 2 from line 513 to line 1012 and so forth).

**CAUTION!** Forgetting the formula of NII in lines without raw data hampers the analysis of the experimental data (Figure 8). Do not forget to delete the Xs when using Excel!

**7.** Evaluation of data is performed through a broad **statistical analysis** (columns R-AH). This analysis shows the number and percentage of nuclei in each group for each experiment (columns R-Z, lines 1-33), as well as for all experiments combined (columns AB-AF, lines 1-13). The averages of area and NII for nuclei in every category of each experiment are also presented (columns Q-AF, lines 37-52). These averages are shown in the graph as closed squares.

**CAUTION!** Don’t change any formula or value on columns starting at AL.

1. We calculate two different roundness measurements, one considering the border perimeter and another considering the perimeter of the correspondent ellipse. By comparison with IPP, we concluded that border perimeter is the one used by IPP to calculate roundness. [↑](#footnote-ref-2)
